# Structure of autoinhibited Akt1 reveals mechanism of PIP_3_-mediated activation

**DOI:** 10.1073/pnas.2101496118

**Published:** 2021-08-12

**Authors:** Linda Truebestein, Harald Hornegger, Dorothea Anrather, Markus Hartl, Kaelin D. Fleming, Jordan T. B. Stariha, Els Pardon, Jan Steyaert, John E. Burke, Thomas A. Leonard

**Affiliations:** ^a^Department of Structural and Computational Biology, Max Perutz Labs, Vienna BioCenter, 1030 Vienna, Austria;; ^b^Department of Medical Biochemistry, Medical University of Vienna, 1090 Vienna, Austria;; ^c^Mass Spectrometry Core Facility, Max Perutz Labs, Vienna BioCenter, 1030 Vienna, Austria;; ^d^Department of Biochemistry and Microbiology, University of Victoria, Victoria, BC V8W 2Y2, Canada;; ^e^Structural Biology Brussels, Vrije Universiteit Brussel (VUB), 1050 Brussels, Belgium;; ^f^VIB-VUB Center for Structural Biology, Vlaams Instituut voor Biotechnologie (VIB), 1050 Brussels, Belgium;; ^g^Department of Biochemistry and Molecular Biology, The University of British Columbia, Vancouver, BC V6T 1Z3, Canada

**Keywords:** kinase, signaling, lipid, PIP3, Akt

## Abstract

The protein kinase Akt is one of the primary effectors of growth factor signaling in the cell. Akt responds specifically to the lipid second messengers phosphatidylinositol-3,4,5-trisphosphate [PI(3,4,5)P_3_] and phosphatidylinositol-3,4-bisphosphate [PI(3,4)P_2_] via its PH domain, leading to phosphorylation of its activation loop and the hydrophobic motif of its kinase domain, which are critical for activity. We have now determined the crystal structure of Akt1, revealing an autoinhibitory interface between the PH and kinase domains that is often mutated in cancer and overgrowth disorders. This interface persists even after stoichiometric phosphorylation, thereby restricting maximum Akt activity to PI(3,4,5)P_3_- or PI(3,4)P_2_-containing membranes. Our work helps to resolve the roles of lipids and phosphorylation in the activation of Akt and has wide implications for the spatiotemporal control of Akt and potentially lipid-activated kinase signaling in general.

## Significance

Akt is an essential protein kinase that controls cell growth, survival, and metabolism. Akt is activated by the lipid second messengers PIP_3_ and PI(3,4)P_2_ and by phosphorylation. However, the relative contributions of lipid binding and phosphorylation to Akt activity in the cell are controversial. Here, we have determined the structure of autoinhibited Akt1, which reveals how the lipid-binding PH domain maintains the kinase domain in an inactive conformation in the absence of PIP_3_. Despite stoichiometric phosphorylation, Akt adopts an autoinhibited conformation with low basal activity in the absence of PIP_3_. Our work reveals the mechanistic basis of Akt hyperactivation in cancer and overgrowth diseases and unambiguously establishes that Akt depends on lipids for activity in the cell.

Akt is a serine/threonine protein kinase essential for cell growth, proliferation, and metabolism (1, 2). Growth factor and insulin signaling via receptor tyrosine kinases activate class I phosphoinositide 3-kinase (PI3K), which generates the lipid second messenger phosphatidylinositol-3,4,5-trisphosphate [PI(3,4,5)P_3_]. Akt is activated by PIP_3_ in the membrane and phosphorylates up to 100 substrates in diverse physiological processes (3). Hyperactivation of the PI3K-Akt pathway is frequently observed in human cancers and overgrowth disorders (4), while autosomal dominant inactivation of Akt2 leads to insulin resistance and diabetes (5).

Akt comprises an N-terminal lipid-binding PH domain with specificity for PI(3,4,5)P_3_ and phosphatidylinositol-3,4-bisphosphate [PI(3,4)P_2_] (6–8) and a C-terminal AGC kinase domain characterized by the presence of a regulatory tail at its C terminus (9). The C-tail of human Akt1 contains two phosphorylation sites, one in the turn motif (T450) and one in the hydrophobic motif (S473). Phosphorylation of the turn motif occurs cotranslationally (10), protects Akt from ubiquitin-mediated degradation (10, 11), and is unaffected by growth factor signaling (11). Recruitment of Akt to PI(3,4,5)P_3_ in the plasma membrane promotes its phosphorylation by phosphoinositide-dependent kinase 1 (PDK1) in its activation loop (T308) (12, 13) and by mTORC2 in its hydrophobic motif (14), rendering it active against substrates. S473 phosphorylation promotes a disorder-to-order transition of the hydrophobic motif (15) that, in synergy with activation loop phosphorylation and ATP, stabilizes a phosphatase-resistant active conformation (16–20) that exhibits catalytic activity orders of magnitude higher than unphosphorylated Akt (21, 22). Akt lacking phosphorylation of these sites is catalytically inactive (23).

Akt exists in an autoinhibited conformation in the cytosol of unstimulated cells, characterized by an intramolecular interaction between its PH domain and the substrate-binding cleft of its kinase domain (20, 23–26). Crystal structures of C-terminally truncated Akt1 in complex with various allosteric inhibitors reveal an interface of ∼1,500 Å^2^ of buried surface area (27–29). Stoichiometric phosphorylation of the activation loop in combination with a phosphomimetic aspartate substitution in the hydrophobic motif is insufficient to overcome the requirement for PI(3,4,5)P_3_ or PI(3,4)P_2_ for full activity (20). Accordingly, imaging of the Akt-substrate complex in live cells revealed that Akt is active only in its membrane-bound state (23). Within the cell interior, PI(3,4)P_2_ has been shown to control Akt signaling on endosomal membranes (23, 30, 31).

Whether PIP_3_ or PI(3,4)P_2_ is necessary for sustained Akt activity in the cell, however, is still controversial. It was recently reported that Akt could not be activated by PI(3,4,5)P_3_ in vitro but that it could be activated independently of lipids by phosphorylation of its hydrophobic motif (21). The authors proposed that phosphorylation of S473 activates Akt through the formation of an electrostatic interaction with a conserved basic residue (R144) in the PH-kinase domain linker, thereby relieving PH domain-mediated autoinhibition. More recently, the mechanism by which S473 phosphorylation induces PH domain displacement and, thereby, Akt activation was reported to rely on a conformational change in the PH domain (32). In this model, Akt can be activated by phosphorylation, independent of its binding to PI(3,4,5)P_3_ or PI(3,4)P_2_. The respective roles of PIP_3_ and phosphorylation in the control of Akt activity are therefore an open question.

In order to resolve the molecular mechanisms of Akt activation, we have determined the high-resolution structure of Akt1 in complex with a nanobody, detailing the autoinhibitory interface between the PH and kinase domains. This interface both sequesters the PIP_3_-binding site of the PH domain and is mutually exclusive with the active conformation of the kinase domain. Rare mutations of Akt associated with cancer (33) and overgrowth disorders (34–36) map to this autoinhibitory interface. By combining a spectrum of biophysical, structural, and biochemical techniques, we demonstrate that, while stoichiometric activation loop and hydrophobic motif phosphorylation increase the basal kinase activity of Akt1 in vitro, they do not completely relieve autoinhibition by its PH domain. Our findings provide clear evidence that full activation of Akt is dependent on both phosphorylation and lipid binding and, therefore, that its activity against cellular substrates is most likely restricted to membranes containing either PI(3,4,5)P_3_ or PI(3,4)P_2_.

## Results

### The PIP_3_-Binding Site Is Sequestered in Autoinhibited Akt.

While structures of Akt in complex with various allosteric inhibitors have provided evidence for an autoinhibitory interaction between the PH and kinase domains (20, 21, 23, 32), the extent to which these inhibitor-bound complexes reflect the physiological conformation and regulation of Akt is still controversial. In order to resolve these issues, we set out to determine the structure of Akt1 without the use of inhibitors. We employed our previously characterized Akt1^DrLink^ (20), which was optimized to encode the shortest evolutionarily tolerated interdomain linker and to avoid heterogeneous phosphorylation of nonconserved residues during heterologous overexpression. We have previously shown that this “DrLink” construct exhibits no discernible differences from wild-type Akt1 with respect to activation by PIP_3_, kinase activity, and membrane binding (20). All constructs analyzed in this study are derivatives of Akt1^DrLink^. However, for reasons of clarity, we use the numbering of human Akt1^WT^ throughout this manuscript, although there is an offset in the kinase domain of Akt1^DrLink^ of -7 amino acids due to the shorter PH-kinase linker. We also omit “DrLink” from the construct nomenclature for reasons of simplicity and clarity. Akt1^DrLink^ is instead designated as Akt1^1P^ by virtue of its stoichiometric turn motif phosphorylation (T450). An overview of all constructs can be found in *SI Appendix*, Fig. S1.

**Fig. 1.**
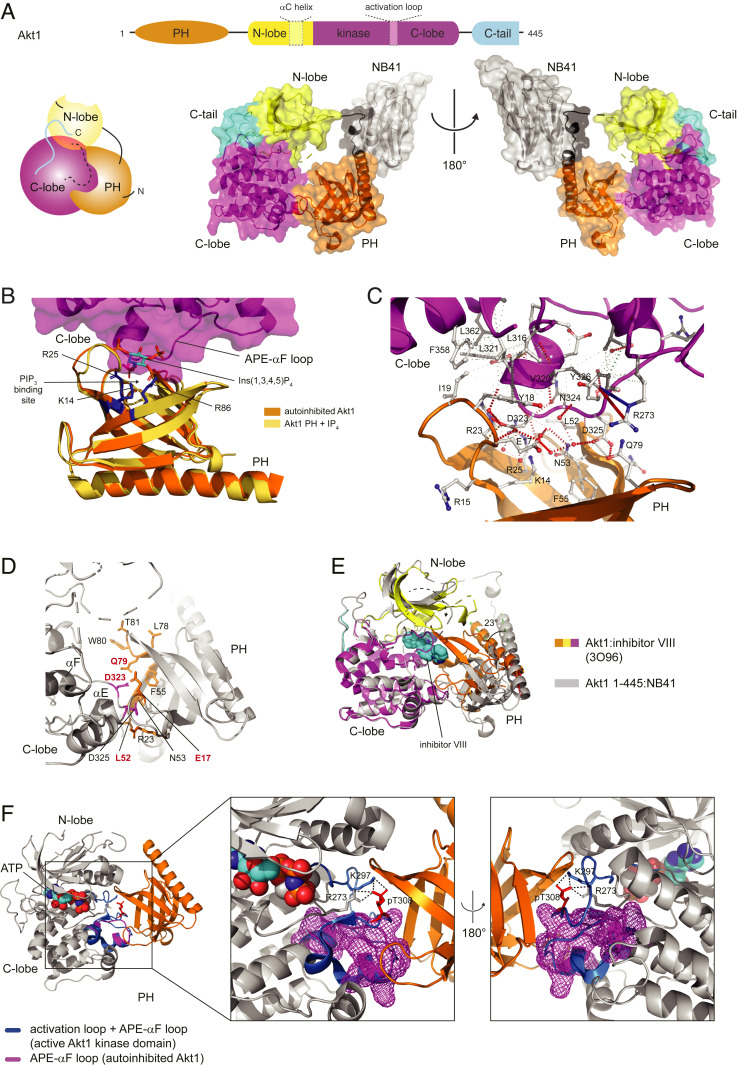
The PIP_3_ binding site is sequestered in autoinhibited Akt. (*A*) Structure of autoinhibited Akt1 1 to 445 in complex with a nanobody. Cartoon schematic illustrates domain architecture of Akt1. Color scheme: PH domain, orange; *N*-lobe of kinase domain, yellow; C-lobe of kinase domain, magenta; C-tail of kinase domain, cyan. Dashed boxes indicate regions of disorder in the structure. (*B*) Superposition of Akt1 PH domain in complex with Ins(1,3,4,5)P_4_ (PDB 1UNQ) with autoinhibited Akt1. Ins(1,3,4,5)P_4_ shown in sticks. PIP_3_-coordinating residues of PH domain shown in blue sticks (3′ phosphate, K14 and R25; 4’ phosphate, R86). (*C*) Interaction map of PH-kinase domain interface. Figure produced using Arpeggio (63). Dashed red lines, hydrogen bonds; red springs, cation-pi; blue springs, donor-pi; gray springs carbon-pi; dotted gray lines, hydrophobic van der Waals. (*D*) Map of disease-associated mutations (red, bold) and mutations that drive growth factor-independent cell survival in vitro (black). Mutations in PH domain shown in orange sticks; mutations in kinase domain shown in magenta sticks. (*E*) Superposition of structure of Akt1 in complex with inhibitor VIII (3O96) on autoinhibited Akt1. Inward rotation of PH domain indicated by 23° rotation of α1. Rmsd of PH domain over all atoms is 9 Å. (*F*) Superposition of active Akt1 kinase domain (4EKK) on autoinhibited Akt1. APE-αF loop of autoinhibited Akt1 shown as magenta mesh. Activation loop and APE-αF loop of active Akt1 shown in blue. Zoom: conformation of phosphorylated T308 in activation loop (red sticks) and network of stabilizing interactions.

Using a llama-derived nanobody as a crystallization chaperone, we determined the structure of near-full length Akt1 (residues 1 to 445) to 2.05 Å resolution (Fig. 1*A* and *SI Appendix*, Fig. S2 *A*–*D* and Table S1). The structure reveals an autoinhibitory assembly in which the entire PIP_3_ binding site on the PH domain is sequestered in an intramolecular interface with a region of extended polypeptide chain between the APE motif of the activation loop and helix αF of the kinase domain, which we refer to as the APE-αF loop (Fig. 1*B*). The interface surface area of 507 Å^2^ is stabilized by one polar cluster involving seven ordered water molecules and one hydrophobic core (Fig. 1*C*): D323 to D325 of the kinase domain interact with K14, R23, R25, N53, and Q79 of the PH domain, and L316, V320, L321, F358, and L362 of the kinase domain associate with Y18 and I19 of the PH domain. We have previously observed that mutation of interfacial residues D323 and D325 leads to PIP_3_ independent activity, increased PIP_3_ and protein substrate affinity, and disruption of the PH-kinase interface, consistent with the critical roles of these residues in maintaining an inhibited conformation (20, 23).

An additional interaction is provided by a cation-pi interaction between Y326 and R273 of the kinase domain, which positions R273 in hydrogen-bonding distance to T82 of the PH domain. The nanobody, which we refer to as NB41, binds to a short sequence of the engineered interdomain linker (Fig. 1*A* and *SI Appendix*, Fig. S2*E*), thereby stabilizing the linker conformation and mediating essential crystal lattice contacts. Previously reported mutations in Akt1, Akt2, and Akt3 associated with Proteus Syndrome, several cancers, and megalencephalies (33–38) and mutations in Akt1 that drive growth factor-independent cell survival in vitro (39) all map to the autoinhibitory interface (Fig. 1*D*). In our structure, the PH domain is rotated ∼23° with respect to the structure of Akt in complex with allosteric inhibitors (27–29), leading to an overall root mean square deviation of 9.04 Å over the whole PH domain (Fig. 1*E* and *SI Appendix*, Fig. S2*F*), indicating that allosteric inhibitors severely distort the autoinhibitory interaction between the PH and kinase domains. In the absence of the C-terminal 35 amino acids, which comprise the turn motif (T450) and the hydrophobic motif (S473), density was not observed for the glycine-rich loop (residues 154 to 157), αC helix (residues 180 to 196), or the activation loop (residues 289 to 306), though inspection of the structure reveals that there are no barriers to the αC helix or the glycine-rich loop from adopting their known physiological conformations in the presence of the C terminus. Superposition of the structure of Akt in the active conformation bound to a substrate peptide and a nonhydrolyzable analog of ATP (PDB 4EKK) with the kinase domain of autoinhibited Akt1 reveals that the conformation of the phosphorylated activation loop and substrate binding are incompatible with the autoinhibitory interface (Fig. 1*F*). Activation loop phosphorylation on T308 elicits a number of subtle but critical conformational rearrangements in the APE-αF loop of the kinase domain. These changes accommodate the active conformation of the activation loop and are required for the hydrogen bond network formed between pT308 with R273 in the catalytic loop and K297 in the activation loop (15, 16). The docking of the phosphorylated activation loop to the surface created by the rearranged APE-αF loop results in steric clashes with the PH domain. This raises the obvious question of whether Akt phosphorylation can override autoinhibition by its PH domain.

### Akt1 Prepared by Protein Semisynthesis Lacks a Phosphorylated Turn Motif.

We previously demonstrated that Akt1 stoichiometrically phosphorylated on T308 and bearing a phosphomimetic serine to aspartate mutation in its hydrophobic motif was still autoinhibited by its PH domain (20). A recent study, however, has suggested that S473 phosphorylation could activate Akt independently of lipids (21). Since phosphorylated serine and aspartate differ both in their chemical makeup and charge, we sought to prepare Akt1 stoichiometrically phosphorylated on T308 (activation loop), T450 (turn motif), and S473 (hydrophobic motif).

**Fig. 2.**
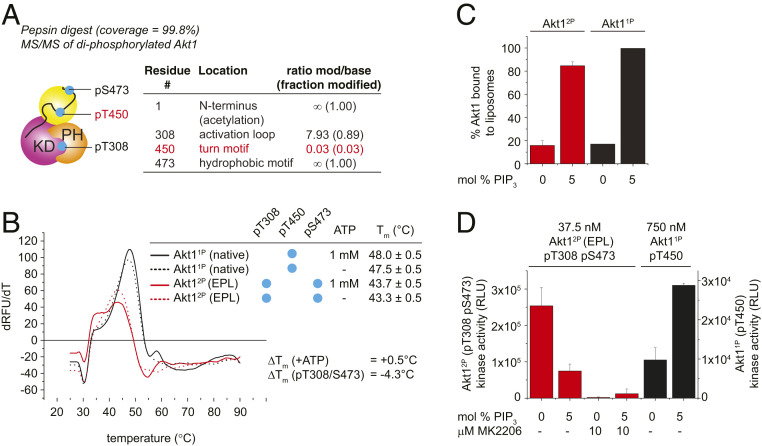
Akt1 prepared by protein semisynthesis lacks a phosphorylated turn motif. (*A*) Phosphorylation state analysis of Akt1 after expressed protein ligation and in vitro phosphorylation with PDK1. Tandem mass spectrometry of pepsin digest. Missing turn motif phosphorylation highlighted in red. (*B*) Thermal stability analysis of Akt1 by differential scanning fluorimetry. Black curves, monophosphorylated Akt1^1P^; red curves, diphosphorylated Akt1^2P^. Solid lines, +1 mM ATP; dashed lines, no ATP. EPL, expressed protein ligation. (*C*) Liposome pelleting assay of Akt1^2P^ and Akt1^1P^ in the presence of 0% and 5% PIP_3_-containing liposomes. (*D*) Akt1 kinase assay in the presence of liposomes containing 0 or 5 mol % PI(3,4,5)P_3_, ± 10 μM MK-2206 (added postliposome binding). Diphosphorylated (T308/S473) Akt1^2P^, red bars; Akt1^1P^, black bars.

Akt1^1P^ purified from baculovirus-infected insect cells is phosphorylated stoichiometrically on T450 but substoichiometrically on T308 (<5%) and S473 (<0.5%) (20). To generate site-specifically phosphorylated Akt, we therefore employed the elegant method of expressed protein ligation previously reported for Akt1 by Chu et al. (21) (*SI Appendix*, Fig. S3*A*). Phosphorylation of T308 was achieved by in vitro incubation with recombinant, active PDK1. The phosphorylation state of Akt1 was monitored by high-resolution anion-exchange chromatography and mass spectrometry. Attempts to prepare tris-phosphorylated Akt1 according to the protocol of Chu et al. resulted in a diphosphorylated species (*SI Appendix*, Fig. S3*B*), in which T308 and S473 were 90% and 100% phosphorylated, respectively, but T450 was less than 3% phosphorylated (Fig. 2*A*). This residual, low-level phosphorylation, may explain why Chu et al. could confirm T450 phosphorylation by Western blotting (21), but mass spectrometry indicates that it is almost completely absent. Henceforth, we refer to this protein as Akt1^2P^ (diphosphorylated on T308 and S473). Since truncation of the C-terminal tail of Akt1 by 24 amino acids in the context of a nonintein fusion protein does not per se affect T450 phosphorylation (*SI Appendix*, Fig. S3*C*), which occurs normally during heterologous overexpression, it seems likely that the presence of the folded *Mxe* GyrA intein domain (21 kDa) just nine amino acids C-terminal to T450 interferes with its phosphorylation. Efforts to solve this problem by moving the ligation site nine amino acids further toward the C terminus (and thereby away from the phosphorylation site) resulted in a fusion protein that was prematurely hydrolyzed during protein expression. The uncleaved protein product, bearing the intein and chitin-binding domain (CBD), was observed to be unphosphorylated, while the cleaved protein product (Akt1 1 to 462) was observed to be stoichiometrically phosphorylated (*SI Appendix*, Fig. S3*D*) on T450. This confirms that it is indeed the intein domain that interferes with the phosphorylation of T450. We observed that loss of pT450 results in a 4.3 °C loss of thermal stability of Akt1 (Fig. 2*B*). In contrast to Akt1^WT^ and Akt1^1P^, which are activated by PI(3,4,5)P_3_-containing liposomes in vitro (20, 23), Akt1^2P^ was actually inhibited by ∼70% upon PI(3,4,5)P_3_ binding (Fig. 2 *C* and *D*). It seems reasonable to conclude that loss of turn motif phosphorylation and consequent destabilization of Akt1 has a negative impact on the behavior of the hydrophobic C-tail in the context of membrane-bound Akt. Attempts to revise the chemical ligation strategy to ligate a C-terminal peptide comprising residues 446 to 480, diphosphorylated on T450 and S473, to Akt1 were in vain, since it was not possible to synthesize the corresponding peptide.

### Phosphorylation Does Not Override the Requirement for PIP_3_.

**Fig. 3.**
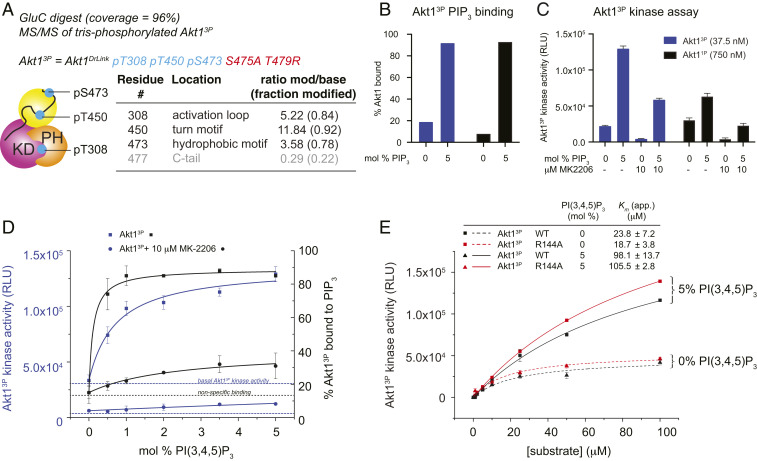
Phosphorylation does not override the requirement for PIP_3_. (*A*) Phosphorylation state analysis of Akt1^3P^ after coculture with A-443654 and okadaic acid. Tandem mass spectrometry of GluC digest. Additional substoichiometric phosphorylation of S477 in 4P species indicated in red. (*B*) Liposome pelleting assay for 0% and 5% PIP_3_ liposomes indicating the binding of Akt1^3P^ and Akt1^1P^ to PIP_3_ in the kinase assay shown in *F*. (*C*) Akt1 kinase assay ± PIP_3_ liposomes, ± 10 μM MK-2206. Akt1^3P^ (37.5 nM), black bars; Akt1^1P^ (750 nM), gray bars. (*D*) Kinase assay of Akt1^3P^ in the presence of liposomes containing increasing concentrations of PIP_3_. *Left* axis, and blue lines correspond to kinase activity. *Right* axis and black lines correspond to % PIP3 binding (determined by a liposome pelleting assay). Squares, Akt1^3P^; circles, Akt1^3P^ preincubated for 10 min with 10 μM MK-2206. PIP_3_ binding and PIP_3_-dependent increase in kinase activity were fit to one-site binding models, taking into account basal Akt1^3P^ activity and nonspecific binding in the presence of 0 mol % PIP_3_ liposomes respectively. (*E*) Kinase assay of Akt1^3P^ and Akt1^3P^ R144A with increasing substrate concentration in the presence of liposomes containing 0 or 5 mol% PIP_3_. Error bars indicate the SD of three independent measurements.

In order to solve the problem of generating stoichiometrically phosphorylated Akt1, we employed a combination of inhibitors to drive hyperphosphorylation of Akt1^1P^ during heterologous expression (*SI Appendix*, Fig. S4*A*). The Akt inhibitor A-443654 has been shown to drive paradoxical hyperphosphorylation of Akt in cells (40, 41). Okadaic acid is a PP2A and PP1 inhibitor that drives global hyperphosphorylation by inhibiting the major cellular phosphatases. Two additional mutations were introduced into Akt1^1P^ to avoid spurious, inhibitor-induced hyperphosphorylation of the C-tail: S475A and T479R. These mutations were designed by inspecting a sequence alignment of Akt isoforms and orthologs, which indicated that these residues are not conserved. Ser475 is hypervariable outside of chordates and is solvent exposed in the structure of active Akt1, while T479 is hypervariable across all Akt orthologs and isoforms. Akt1 prepared in this manner exhibited hyperphosphorylation of up to seven sites, of which T308, T450, and S473 were the most abundant modifications. Modification of S123, T127, and S132 in the PH-kinase linker and S477 in the C-terminal tail were found in the 7-phospho (7P) species (*SI Appendix*, Fig. S4*B*), but not in the tris-phosphorylated species (3P). Tris-phosphorylated Akt1 was isolated by high-resolution anion-exchange chromatography and verified by intact mass spectrometry (*SI Appendix*, Fig. S4*C*). The stoichiometric phosphorylation of this protein on T308, T450, and S473 was confirmed by tandem mass spectrometry (Fig. 3*A*). Henceforth, we refer to this protein as Akt1^3P^.

We first compared the thermal stability of Akt1^3P^ to Akt1^2P^ and Akt1^1P^ (1P). We observed comparable thermal stabilities between Akt1^3P^ and Akt1^1P^, but a 4 °C loss of stability in Akt1^2P^ (*SI Appendix*, Fig. S4*D*) as previously observed (Fig. 2*B*). Since Akt1^2P^ differs from Akt1^1P^ and Akt1^3P^ by the phosphorylation of T450, we concluded that absent T450 phosphorylation adversely affects the stability of Akt1. We next subjected Akt1^3P^ to a kinase assay in the presence of liposomes with or without PI(3,4,5)P_3_ incorporation. We first established PIP_3_ binding by performing a liposome pelleting assay (Fig. 3*B*) before determining kinase activity on the same sample in the presence and absence of the allosteric inhibitor MK-2206 (Fig. 3*C*). Akt1^3P^ was robustly activated by liposomes containing 5 mol % PI(3,4,5)P_3_ and inhibited by MK-2206, though the efficacy of MK-2206 was significantly attenuated by PIP_3_ binding (Fig. 3*C*). As a control, we used Akt1^1P^ (20), though a 20-fold lower concentration of Akt1^3P^ was required due to the higher basal activity of Akt1^3P^ in the absence of liposomes containing PIP_3_ compared to Akt1^1P^. In order to confirm that Akt1^3P^ is indeed activated by PI(3,4,5)P_3_ in a concentration-dependent manner, we performed a kinase assay in the presence of liposomes containing increasing amounts of PI(3,4,5)P_3_. As expected, Akt1^3P^ was activated in a concentration-dependent manner that mirrors its binding to the liposomes. Preincubation of Akt1^3P^ with the Akt-specific inhibitor MK-2206 almost completely abolished the increase in activity (Fig. 3*D*), confirming that the observed activity and PIP_3_-dependent increase in activity is attributable to Akt1^3P^. Preincubation of Akt1^3P^ with MK-2206 prevented PIP_3_-mediated membrane binding (Fig. 3*D*), consistent with its sequestration of the PH domain in a PIP_3_-inaccessible conformation.

It has previously been claimed that R144 in the PH-kinase linker plays a crucial role in activating Akt1 by coordinating phosphorylated S473 in the hydrophobic motif. Mutation of R144 to alanine was reported to decrease catalytic activity 50-fold (21). However, given that Akt1 stoichiometrically phosphorylated on S473 is still activated by PIP_3_ and intein-mediated protein ligation gives rise to Akt1 lacking turn motif phosphorylation, we next tested whether R144 in the PH-kinase linker indeed influences kinase activity. We first prepared stoichiometrically phosphorylated Akt1^3P^ R144A according to the same procedure detailed in *SI Appendix*, Fig. S4*A*. Mass spectrometry confirmed phosphorylation of T308, T450, and S473 (*SI Appendix*, Fig. S4*E*). However, mutation of R144 had no effect on kinase activity either in the presence or absence of PIP_3_, while both proteins were activated equally (Fig. 3*E*).

In summary, while phosphorylation increases the basal kinase activity of Akt1 in vitro, under conditions of saturating substrate concentration, it is not sufficient to override autoinhibition by its PH domain.

### Phosphorylation Alone Does Not Drive Akt into an Active Conformation.

**Fig. 4.**
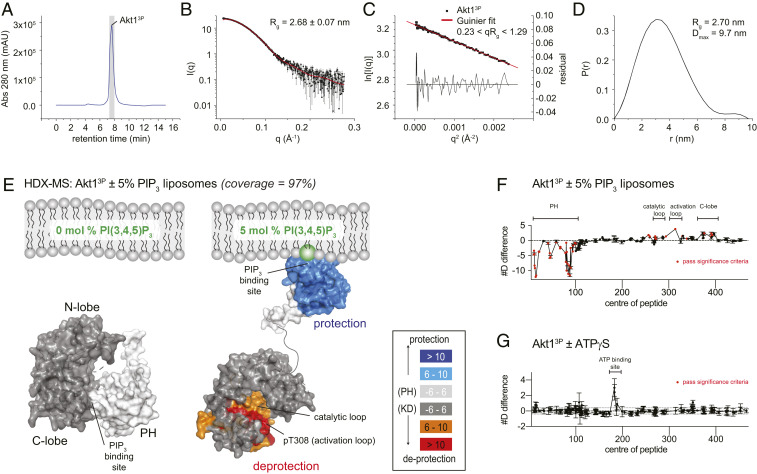
Phosphorylation alone does not drive Akt into an active conformation. (*A*) Size-exclusion profile of Akt1^3P^ from in-line SEC-SAXS data collection. Gray bar indicates the region of the chromatogram evaluated in the SAXS data processing. (*B*) SAXS curve of Akt1^3P^. Radius of gyration (R_g_) derived from Guinier analysis of the low-angle scattering regime. (*C*) Guinier plot of the low-angle SAXS regime for Akt1^3P^. (*D*) Pair distribution function for Akt1^3P^, indicating the radius of gyration (R_g_) and maximum dimension of the particle (D_max_). (*E*) Hydrogen-deuterium exchange mass spectrometry analysis of Akt1^3P^ in the presence of liposomes containing 0 or 5 mol % PI(3,4,5)P_3_. Regions of Akt1^3P^ that showed significant increases or decreases in exchange (meeting the three criteria: ≥6% change in exchange, ≥0.4 Da difference in exchange, and a *P* value <0.01 using a two-tailed Student’s *t* test) upon PIP_3_ binding are mapped on the structures of the PH domain and the active kinase domain (PDB ID 4EKK) (16) with the corresponding color scheme. (*F*) Plot of differences in deuterium incorporation upon PIP_3_ binding. Changes in deuterium incorporation are plotted against the center of each peptide. Regions of protection and deprotection are indicated above the plot and correspond to those mapped in *E*. Error bars indicate the SD of three independent replicates. Red data points indicate increases or decreases in exchange that passed the three significance criteria. (*G*) Plot of changes in deuterium incorporation upon ATPγS binding. Changes in deuterium incorporation are plotted against the center of each peptide. Regions of protection and deprotection are indicated above the plot. Error bars indicate the SD of three independent replicates. No changes were deemed significant according to the three significance criteria.

The dependency of tris-phosphorylated Akt1 on PIP_3_ for full activity strongly implied that Akt1 is autoinhibited by its PH domain in the presence of T308 and S473 phosphorylation. To support this conclusion, we collected small-angle X-ray scattering (SAXS) data on Akt1^3P^ in solution and compared the particle parameters to those previously obtained for Akt1^WT^, Akt1^1P^, and Akt1^DA^ (20). Akt1^DA^ bears two mutations at D323 and D325 located in the autoinhibitory PH-kinase interface and exhibits both an open conformation and PIP_3_-independent kinase activity (20, 23). To avoid any protein aggregates in our SAXS analysis we employed in-line size exclusion chromatography (Fig. 4*A*). The radius of gyration of Akt1^3P^ was estimated by Guinier analysis of the low-angle portion of the scattering curve (Fig. 4 *B* and *C*) and by calculation of the pair-distribution function (Fig. 4*D*), which estimates the maximum dimension of the particle, D_max_. Akt1^3P^ exhibits identical values for R_g_ (2.63 to 2.70 nm) and D_max_ (9.7 nm) to Akt1^WT^ and Akt1^1P^ and is significantly more compact than the open conformation of Akt1^DA^ (R_g_ = 3.1 nm, D_max_ = 12.2 nm) (20). These data indicate that stoichiometric phosphorylation of Akt1 does not induce a conformational change consistent with displacement of its PH domain.

To directly observe the conformational changes in Akt1^3P^ elicited by PIP_3_ binding, we performed hydrogen-deuterium exchange mass spectrometry (HDX-MS) analysis of Akt1^3P^ in the presence of liposomes containing either 0% or 5% PIP_3_. The sequence coverage of Akt1 was excellent, spanning ∼97% of all exchangeable amides (*SI Appendix*, Table S2). With 5% PIP_3_ liposomes, there was extensive protection of the PH domain, similar to what had been previously observed for the PH domain of nonphosphorylated Akt1 (20). In addition to protection of the PH domain elicited by PIP_3_ binding, Akt1^3P^ exhibited significant deprotection of the C-lobe of the kinase domain, including the activation loop, that corresponds to the interface between the PH and kinase domains observed in our structure of autoinhibited Akt1 (Fig. 4 *E* and *F* and *SI Appendix*, Fig. S5*A*). We also carried out HDX-MS experiments in the presence and absence of ATPγS, for which we observed no significant differences (Fig. 4*G*). This indicates that ATP binding (23) does not drive tris-phosphorylated Akt1^3P^ into an open conformation in the absence of PIP_3_.

### PIP_3_ Binding Exposes the Hydrophobic Motif of Akt.

**Fig. 5.**
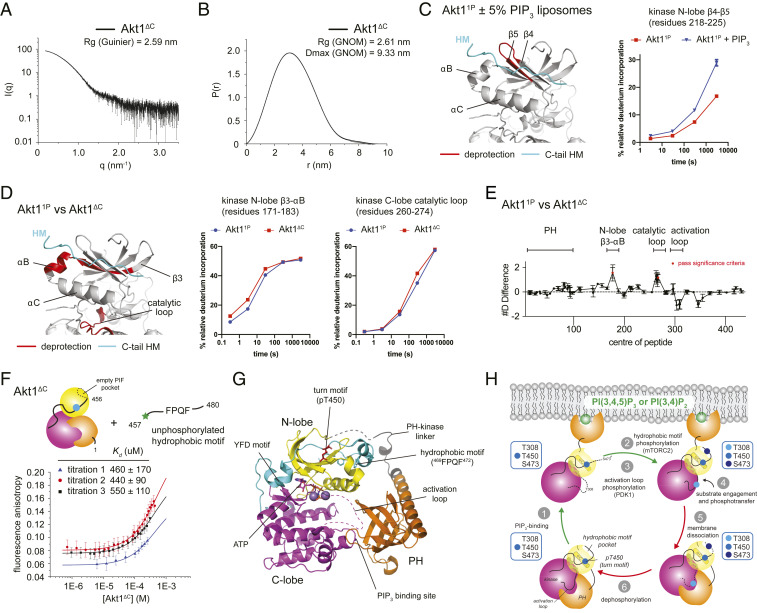
PIP_3_ binding promotes Akt hydrophobic motif exposure. (*A*) SAXS scattering curve for Akt1^ΔC^. (*B*) Pair-distribution function (PDF) for Akt1^ΔC^ indicating the radius of gyration (R_g_) and maximum dimension of the particle (D_max_). (*C*) HDX-MS of Akt1^1P^ ± PIP_3_ liposomes. Exposure (deprotection) of *N*-lobe peptide 218 to 225 indicated in red on structure of kinase domain. Plot: deuterium incorporation as a function of time for Akt1^1P^ ± PIP_3_ liposomes. Deuterium incorporation plots were reproduced with permission. Adapted from ref. 20, which is licensed under CC BY-NC-ND 4.0. (*D*) HDX-MS of Akt1^1P^ versus Akt1^ΔC^. Two regions showed significant increases in exchange (meeting the three criteria: ≥6% change in exchange, ≥0.4 Da difference in exchange, and a *P* value <0.01 using a two-tailed Student's *t* test). Regions 171 to 183 (YAMKILKKEVIVA) in the *N*-lobe and 260 to 274 (HSEKNVVYRDLKLEN) in the C-lobe are indicated in red on the structure of the kinase domain. Deuterium incorporation plots for these peptides as a function of time for Akt1^1P^ and Akt1^ΔC^ are shown to the right. (*E*) Plot of changes in deuterium incorporation between Akt1^1P^ and Akt1^ΔC^. Changes in deuterium incorporation are plotted against the center of each peptide. Regions of protection and deprotection are indicated above the plot and correspond to those mapped in *D*. Error bars indicate the SD of three independent replicates. Red data points indicate increases or decreases in exchange that passed the three significance criteria. (*F*) Fluorescence anisotropy binding assay for C-terminal tail peptide (FITC-SMEAVDSERRPHFPQFSYSASGTA) to Akt1^ΔC^. The *K*_D_ was estimated from three independent titrations. Each data point is the mean of 50 technical replicates with an integration time of 1 s. Error bars indicate the SD from the mean. Data were fit to a one-site binding model. (*G*) Composite model of full-length Akt1. The model comprises autoinhibited Akt1 (PH domain, PH-kinase linker, and kinase domain C-lobe), the *N*-lobe of active Akt1 (4EKK), and the phosphorylated C-terminal regulatory tail of PKCι (4DC2). The inactive conformation of the activation loop (unknown) is indicated with dashed magenta lines. (*H*) Stepwise activation of Akt by PIP_3_ and phosphorylation. Activating steps are indicated with green arrows. Inactivating steps are indicated with red arrows. Phosphorylation state of each species in the activation and inactivation cycle is indicated in the blue boxes for each of the three regulatory residues: T308, T450, and S473.

Since it was necessary to delete the C-terminal 35 amino acids of Akt1 to facilitate crystallization, we investigated the conformation and accessibility of the hydrophobic motif in Akt1. We purified Akt1 1 to 456 (Akt1^ΔC^), a C-terminally truncated construct missing the hydrophobic motif but retaining the stabilizing turn motif site. We confirmed that the recombinant protein is stoichiometrically monophosphorylated on T450 in the turn motif (*SI Appendix*, Fig. S3*C*). SAXS analysis revealed that Akt1^ΔC^ adopts a compact conformation in solution with an identical radius of gyration and maximum dimension of the particle as Akt1^WT^ (20) (Fig. 5 *A* and *B* and *SI Appendix*, Fig. S6*A*), indicating that the hydrophobic motif is not required for interaction of the PH and kinase domains. This corroborates our crystal structure of C-terminally truncated Akt1. Analysis of the thermal stability of Akt1^ΔC^ indicates a modest reduction of 1.5 °C compared with full-length Akt1^1P^ (*SI Appendix*, Fig. S6*B*), suggesting that, although not sufficient to destabilize Akt completely, deletion of the hydrophobic motif does in fact weaken the overall structure of Akt. By contrast, however, additional mutation of residues in the interface between the PH and kinase domains (Akt1^DA^ ^ΔC^) destabilizes Akt1 by 8 °C (*SI Appendix*, Fig. S6*B*), just as we have previously observed in the context of full-length Akt1 (20). We confirmed that Akt1^DA^ ^ΔC^ is also stoichiometrically monophosphorylated (*SI Appendix*, Fig. S6*C*). Together, these findings indicate that the inactive conformation of Akt1 does not depend on the hydrophobic motif but that it may actively sequester the hydrophobic motif in a bound conformation.

The likely binding pocket for the hydrophobic motif is the so-called PDK1-interacting fragment (PIF) pocket in Akt that binds the phosphorylated hydrophobic motif in the active conformation (15, 16). Previous HDX-MS analysis of Akt1^1P^ indicated small but significant exposure of the PIF pocket upon PI(3,4,5)P_3_ binding (20) (Fig. 5*C*). We confirmed this observation by comparing the deuterium incorporation rates for Akt1^1P^ and Akt1^ΔC^ in solution. Sequence coverage of the truncated Akt1^ΔC^ comprised 85 peptides spanning ∼94% of all exchangeable amides (*SI Appendix*, Table S2). Two peptides in Akt1^ΔC^, corresponding to β3-αB in the *N*-lobe and the catalytic loop in the C-lobe of the kinase domain, exhibited a modest, but significant, 6% increase in deuterium incorporation (Fig. 5 *D* and *E*). These changes indicate exposure of the PIF pocket and consequent local disordering of the *N*-lobe in the absence of the hydrophobic motif. These observations are consistent with the lack of electron density observed for the αC helix and activation loop and overall higher temperature factors for the *N*-lobe of the kinase domain (*SI Appendix*, Fig. S6*D*). In order to test whether the unphosphorylated hydrophobic motif indeed binds to the PIF pocket in the inactive conformation, we measured the binding affinity of a tail peptide containing the missing C-terminal tail residues in Akt1^ΔC^ (residues 457 to 480 of human Akt1) by fluorescence anisotropy. The binding constant was estimated to be ∼0.5 mM from three independent titrations (Fig. 5*F*), although it was not possible to reach saturation due to limiting Akt1^ΔC^ concentration. While this is a relatively weak interaction, it is sufficient in the context of an intramolecular interaction to sequester the hydrophobic motif more than 99% of the time at equilibrium due to the almost infinite local concentration.

Taken together with the crystal structure of Akt1 1 to 445, we propose a model for the structure and conformation of full-length Akt1 (Fig. 5*G*) in which the C-terminal tail binds in its unphosphorylated state to the PIF pocket of the kinase domain, thereby stabilizing the active conformation of the αC helix and promoting ATP binding. This composite model, which includes an ordered αC helix and C-terminal tail, fits very well into the molecular envelope of Akt1^1P^ previously calculated from SAXS data in solution (20) (*SI Appendix*, Fig. S6*E*).

## Discussion

We present here a structure of autoinhibited Akt1, providing a near-atomic resolution picture of the autoinhibitory interface between its PH and kinase domains. This confirms previously published findings indicating that the PIP_3_-binding pocket is sequestered in the inactive conformation (20, 23, 27). A number of recurrent mutations in Akt have been identified in breast cancer (37), Proteus syndrome (34), and hemimegalencephaly (35). Many of these mutations map to the interface between the PH and kinase domains. Mutation of E17K has been shown to promote membrane binding and cellular transformation in vitro (37) with a corresponding increase in membrane affinity and loss of specificity for PIP_3_ (38). In the structure of autoinhibited Akt1, E17 forms a salt bridge with R86, which stabilizes the loop conformation between strands β1 and β2 that packs against the kinase domain. Substitution of E17 with lysine would cause an energetically unfavorable charge repulsion with R86 and consequent destabilization of the interface. Mechanistically, E17K therefore likely promotes membrane binding and hyperactivation of Akt by lowering the energy barrier to Akt activation by PIP_3_ and shifting the equilibrium to the membrane-bound fraction. This finding is consistent with the destabilizing effect of E17K on the PH-kinase domain interaction observed in a mammalian two-hybrid assay (39) and the observation that E17K is still dependent on PIP_3_ for full activation (23). D323 is one of a pair of invariant aspartates in the APE-αF loop of the kinase domain, mutation of which we have previously shown to promote a conformational change in Akt, uncouple Akt activity from PIP_3_, and promote Akt hyperphosphorylation (20, 23). D323 and D325 form a network of hydrogen bonds with residues from the PH domain and seven ordered water molecules, which obscures the PIP_3_ binding site. This explains why mutation of the kinase domain at D323 and D325 or deletion of the kinase domain promotes binding of the PH domain to PIP_3_ in vitro and in vivo (23). Hyperphosphorylation and constitutive activity of Akt1^DA^ is therefore driven by a combination of enhanced membrane association and relief of the inhibitory interface that blocks formation of the active conformation of the activation loop. Although the interface surface area is relatively small at just over 500 Å^2^, it is stabilized by numerous water-mediated hydrogen bonds that have previously been shown to be critical for the association of hydrophilic protein interfaces (42) as well as a set of hydrophobic interactions.

The PH domain of Akt1 interacts with a surface of the kinase domain that fulfils important regulatory functions in a wide range of eukaryotic protein kinases. In protein kinase R, for example, the αG helix as well as the C-terminal part of the activation segment has been shown to function as a docking surface for its substrate eIF2α (43), while the same surface binds the inhibitory regulatory subunit of protein kinase A in the holoenzyme complex (44). In Cdk2, a surface surrounding the αG helix facilitates the binding of the CDK-interacting protein phosphatase KAP to its phosphorylated activation loop, thereby imposing specificity on phosphatase-mediated inactivation (45). Finally, the αG helix mediates heterodimerization of B-Raf and MEK1, critical for B-Raf–mediated MEK1 activation by activation loop phosphorylation (46) as well as, conversely, the autoinhibition of the death-associated protein kinase 2 (DAPK2) (47). In summary, protein–protein interactions mediated by this surface of the C-lobe permit both activation and inhibition of kinase activity as well as specific substrate phosphorylation or even kinase inactivation by phosphatases.

The PH-kinase interface in autoinhibited Akt1 is considerably different to that reported for Akt1 in complex with allosteric inhibitors (27–29). Allosteric inhibitors appear to stabilize a nonnative conformation of Akt by binding to both the PH and kinase domains, an interaction that depends critically on W80 in the β6-β7 loop of the PH domain (24). While W80 mediates a number of contacts that stabilize the inhibitor-bound conformation, W80 does not make any contacts with the kinase domain in our structure. This observation likely reflects the fact that the C-terminal tail is missing and, consequently, the αC helix of the *N*-lobe is disordered. However, it also indicates that W80 is not essential for maintenance of the autoinhibited conformation of the PH and kinase domains and more likely participates in sequestering the *N*-lobe, activation loop, and C-terminal tail in their inactive conformations. It is worth noting that only small changes in the conformation of the activation loop from the active state would be required in order to accommodate it in the space between the PH domain and *N*-lobe of the kinase domain, which would sequester it from phosphorylation in the absence of PIP_3_. Confirmation of this hypothesis will, however, require a structure in which the C terminus and activation loop are fully visualized.

The mutual exclusivity of the inactive and active conformations of Akt defined by their corresponding structures raises the obvious question of whether phosphorylation of the kinase domain can displace the PH domain and thereby override the requirement for PIP_3_. In this respect, a recent study was unable to demonstrate the PIP_3_-mediated activation of site-specifically phosphorylated Akt1 prepared by protein semisynthesis (21). To resolve this issue, we produced Akt1 by the same intein-based protein ligation method reported by Chu et al. (21). By using Akt1^1P^, previously optimized for homogeneous phosphorylation on T308, T450, and S473, we could routinely monitor the phosphorylation state of Akt both by high-resolution anion-exchange chromatography and mass spectrometry. The latter, however, indicated that the ligation product, while stoichiometrically phosphorylated on T308 and S473, was missing T450 phosphorylation. This is consistent with the lack of observed electron density for the phosphorylated turn motif in both crystal structures of the Akt1 kinase domain reported by the study. The consequences of absent turn motif phosphorylation are significantly reduced protein stability and a >70% decrease in kinase activity when bound to PIP_3_, presumably due to the disordered nature of the C-terminal hydrophobic tail in the vicinity of the membrane. The absence of turn motif phosphorylation likely also explains the millimolar *K*_*m*_ values for ATP reported by Chu et al. for most constructs (21). We previously determined the affinity of monophosphorylated (T450) Akt1^1P^ for ATP to be 100 μM (23), while a kinetic study determined the *K*_*m*_ for ATP to be 53 μM (48). The importance of turn motif (T450) phosphorylation in regulating the stability of Akt and other AGC kinases is also well established (10, 11).

PIP_3_-independent activation of Akt1 by S473 phosphorylation has recently been proposed to be mediated by a conserved basic patch in the PH-kinase interdomain linker (21). The interaction of R144 with phosphorylated S473 in the hydrophobic motif was suggested to dislodge the PH domain from the kinase domain by inducing a conformational change in the linker. More recently, a follow-up study has attempted to shed light on the proposed mechanism. Using protein semisynthesis to segmentally label Akt1 for NMR studies, the authors suggest that S473 phosphorylation induces a loop-helix transition of residues 44 to 46 (DVD) in the PH domain, which dislodges it from its autoinhibitory interaction with the kinase domain. However, these findings are very difficult to reconcile with the structure of autoinhibited Akt1 and the critical role of PIP_3_ in PH domain displacement and, consequently, kinase activation. Importantly, analysis of S473-phosphorylated Akt1 with two complementary biophysical techniques (SAXS and HDX-MS) unambiguously shows that S473 phosphorylation does not dislodge the PH domain from the kinase domain. Furthermore, mutation of R144 did not affect the kinase activity of Akt1^3P^ or its activation by PIP_3_ in vitro.

We have shown here that stoichiometrically phosphorylated Akt1 is activated by PIP_3_ in a concentration-dependent manner that corresponds to its binding. We can be confident in this finding for two reasons: first, in order to directly correlate PIP_3_ binding with changes in kinase activity, we subjected half of the kinase reaction to a liposome pelleting assay in order to quantify the degree of PIP_3_ binding, which was then compared with the signal from the kinase assay; secondly, preincubation of Akt1^3P^ with MK-2206, a specific allosteric Akt inhibitor, completely abrogated the observed activity and the PIP_3_-dependent increase in activity. The activation of Akt1^3P^ by PIP_3_ is further supported by the observation that PIP_3_ binding elicits the same conformational changes in Akt1^3P^, as we previously reported for Akt1^1P^ (20). Nevertheless, phosphorylated Akt1 retains significant basal activity, which raises the question of how the active conformation is obtained in the absence of PIP_3_. We suggest that this is the consequence of an equilibrium between open and closed conformations. When phosphorylated, Akt is able to sample the active conformation under conditions in which the PH domain dissociates from its inhibitory interaction with the kinase domain. We previously showed, however, that this equilibrium is heavily biased toward the closed conformation in the absence of PIP_3_ (20). The fact that the PH domain must dissociate from the kinase domain in order to bind PIP_3_ strongly supports the existence of such an equilibrium.

In summary, binding of PI(3,4,5)P_3_ promotes the exposure and phosphorylation of the activation loop (13, 20, 49) as well as exposure of the hydrophobic motif (20), presumably leading to its phosphorylation by mTORC2 in vivo. Consistent with this hypothesis, deletion of the PH domain promotes mTORC2-independent phosphorylation of S473 in the hydrophobic motif of Akt in Sin1^−/−^ mouse embryonic fibroblasts (50). Phosphorylation of T308 and S473, in the context of PIP_3_ binding, results in disorder-to-order transitions of the activation loop and hydrophobic motif, respectively, and the structuring of a highly ordered, high-affinity substrate-binding site as well as the catalytic machinery for phospho-transfer (15, 16). Without phosphorylation, Akt is essentially inactive (23). This explains why two recent studies as well as early enzymatic studies on Akt observed huge increases in *k*_*cat*_ upon dual activation loop and hydrophobic motif phosphorylation, independent of the presence of any lipids (21, 22). In vitro kinase assays are typically done in the presence of large excesses of substrate in order to obtain a robust signal and do not reflect reality in the cell, in which substrate concentrations are orders of magnitude lower. However, scaffolding of protein kinases and their substrates is a well-established mechanism by which effective substrate concentrations can be increased by orders of magnitude. Indeed, Akt has been reported to exist in a complex with PI3K and PDK1, held together by the scaffold protein IQGAP1 (51). Scaffolding of kinases and substrates in this way increases the fidelity and flux of signal transduction.

Lipid binding and phosphorylation synergize to increase the substrate-binding affinity of Akt1 by relieving a steric block to substrate binding, increasing the rate of phospho-transfer by positioning the catalytic machinery correctly, and creating a phosphatase-resistant conformation of Akt on the membrane in an ATP-dependent manner. In this way, Akt is primed for iterative cycles of substrate phosphorylation and signal amplification. By contrast, autoinhibited Akt, irrespective of its phosphorylation state, has a low affinity for substrate with a correspondingly lower activity, and is susceptible to phosphatase-mediated inactivation, which is rate limited by dissociation from PIP_3_ in the cell. This susceptibility to dephosphorylation in the absence of lipids is consistent with the requirement for global phosphatase inhibition by okadaic acid during protein expression to drive Akt1 into a stoichiometrically phosphorylated state (this study). It therefore seems reasonable to conclude that substrate phosphorylation by Akt in the cell strongly depends upon PI(3,4,5)P_3_ or PI(3,4)P_2_. This mechanism (Fig. 5*H*), which involves the coincident detection of either PIP_3_ or PI(3,4)P_2_ and the upstream kinases PDK1 and mTORC2, functions like an electronic logic gate (AND) to restrict Akt activity to locations in the cell where these inputs are present. Coupling Akt activity to both lipids and upstream kinases ensures the fidelity of Akt signaling downstream of PI3K.

Akt is able to phosphorylate many downstream substrates, including both cytosolic and nuclear substrates, with phosphorylation of these molecules likely dependent on the migration of these substrates to membranes containing either PI(3,4)P_2_ or PIP_3_. Multiple studies have identified pools of actively signaling PI(3,4)P_2_ in the cell interior, and evidence for nuclear phosphoinositides is accumulating. Cytosolic Akt activity has previously been reported with the use of genetically encoded kinase activity reporters (52, 53). An earlier study, however, only detected Akt activity with fluorescence-based sensors localized to membrane-bound compartments (54). More recently, intracellular Akt activity has been correlated with significant pools of endomembrane PI(3,4)P_2_ using the same fluorescence-based activity reporters (30). In this respect, fluorescence cross-correlation experiments in live cells did not detect evidence of freely diffusing Akt-substrate complexes in the cytosol (23). While these experiments do not rule out the possibility of cytosolic Akt activity, the evidence suggests that Akt activation and most of its subsequent activity is dependent on PIP_3_ or PI(3,4)P_2_. While it is eminently conceivable that substrates of Akt that exert their functions in the nucleus could be phosphorylated by Akt bound to PI(3,4)P_2_-rich endomembranes in the cytoplasm (55), it should be noted that evidence of nuclear Akt signaling has also recently been obtained (56). Further studies will undoubtedly be required to understand precisely how and under what conditions Akt is activated in the nucleus.

## Materials and Methods

### Protein Expression and Purification.

Akt1 constructs were coexpressed with human PDK1 in baculovirus-infected Sf9 cells using a pFastBac Dual vector. For nanobody screening, biotinylated Akt1^DB^ (biotinylated Akt1) was expressed by fusing the AviTag sequence (GLNDIFEAQKIEWHE) to the N terminus of an unstructured 50 amino acid sequence from tumor susceptibility gene 101 (residues 145 to 194, C188S) and appending this sequence to the N terminus of Akt1. Recombinant baculovirus was prepared from a pFastBac Dual construct expressing Akt1^DB^ in the polyhedrin cassette and *Escherichia coli* BirA in the p10 cassette, and the medium was supplemented with 10 μM D-biotin during protein expression. Detailed protein purification protocols can be found in the *SI Appendix*.

### Mass Spectrometry.

Detailed protocols for intact and tandem mass spectrometry as well as HDX-MS can be found in the *SI Appendix*.

### Nanobody Generation against Akt1^DrLink^.

Akt1^1P^-specific nanobodies were raised by immunizing a llama as previously described (57). Details of the specific protocol can be found in the *SI Appendix*.

### Surface Plasmon Resonance Affinity Measurements.

All surface plasmon resonance experiments were performed using a Biacore T200 and a streptavidin-coated Biacore CAPture chip. To measure the binding kinetics of nanobody NB41, the chip was loaded with 10 µg/mL Akt1^DB^ in 20 mM Tris pH 7.5, 100 mM NaCl, 0.1% BSA, and 0.05% Tween for 2 min at 10 µL/min. Three startup cycles, during which the Akt1^DB^-loaded chip was equilibrated with buffer at 30 µL/min for 2 min, were performed to stabilize the sensor chip surface. The binding kinetics of NB41 were determined via single cycle kinetics (58) by measuring five increasing nanobody concentrations (3.125 nM, 6.25 nM, 12.5 nM, 25 nM, and 50 nM) with association and dissociation intervals of 60 s at a flow rate of 30 µL/min. The chip surface was regenerated and the ligand reloaded after every cycle. *k*_on_ and *k*_off_ rates were determined via curve fitting.

### Crystallization and Structure Determination.

Akt1^1-445^-SR, derived from MESNA-mediated cleavage of Akt1^1-445^-intein-CBD, was combined with purified NB41 at a 1:1.5 ratio and the complex separated from free NB41 by SEC on a Superdex 200 10/30 column. The complex crystallized in 200 mM malonate, pH 5.0, and 16% PEG 3350. Crystals were cryoprotected in mother liquor supplemented with 25% (vol/vol) glycerol and plunge frozen in liquid nitrogen. Crystals grew in spacegroup *P*2_1_2_1_2_1_, with unit cell dimensions *a* = 70.01 Å, *b* = 72.20 Å, and *c* = 120.17 Å (α = β = γ = 90°). Data were collected to 2.05 Å resolution on ID23-2 at the European Synchrotron Radiation Facility (ESRF). The structure was solved by molecular replacement using PHASER (59) with 4EKK (Akt1 kinase domain), 1UNP (Akt1 PH domain), and 3EZJ (nanobody) as input models. The model was built in Coot (60) with iterative rounds of refinement and model validation in PHENIX (61). Data processing and model building statistics are reported in *SI Appendix*, Table S1. The coordinates of Akt1^DrLink^ 1 to 445 have been deposited in the Protein Data Bank with the identifier: 7APJ.

### SAXS.

SAXS data for Akt1^ΔC^ and Akt1^3P^ were collected on BM29 at the ESRF, Grenoble, France using an in-line SEC-SAXS setup as described in Lučić et al. (20). Proteins were applied to a Superdex 200 column equilibrated in 20 mM Tris, pH 7.4, 100 mM NaCl, 1 mM DTT, and 1% (vol/vol) glycerol and images were acquired every second for the duration of the size exclusion run. Buffer subtraction was performed by averaging 50 frames either side of the peak. All subsequent data processing steps were performed using the ATSAS data analysis software 3.9.1. The program DATGNOM (62) was used to generate the pair-distribution function [P(r)] for each isoform and to determine Dmax and Rg from the scattering curves [I(q) versus q] in an automatic, unbiased manner.

### Preparation of Sucrose-Loaded Vesicles and Liposome Pelleting Assay.

Liposomes and liposome pelleting assays with Akt were performed as previously reported (20).

### Akt1 Kinase Assays.

Kinase assays were performed according to the Promega ADP-Glo protocol. In brief, upon binding of Akt1 proteins (Akt1^1P^, Akt1^2P^, or Akt1^3P^) to PI(3,4,5)P_3_–containing vesicles, the protein/vesicle mixtures were incubated with Crosstide (GenScript) and ATP/MgCl_2_ with or without Akt inhibitor MK-2206 for 1 h at RT. The assay contained a final concentration of 100 μM ATP, 200 μM MgCl_2_, 100 μM Crosstide, and 750 nM Akt1^1P^, 37.5 nM Akt1^2P^, or 37.5 nM Akt1^3P^ with or without 5 to 10 μM MK-2206. Luminescence was read out in a TECAN 500 infinite plate reader. For end-point assays, 10 μM MK-2206 was added postliposome binding; for PIP3 titration, Akt1^3P^ was preincubated with 20 μM MK-2206 for 20 min prior to addition to the liposomes (10 μM MK-2206 final).

### Thermal Stability Assays.

The thermal stabilities of Akt1^1P^, Akt1^2P^, Akt1^3P^, Akt1^ΔC^, and Akt1^DA^ ^ΔC^ were measured by differential scanning fluorimetry. Samples contained 0.2 mg/mL protein in 25 mM Tris pH 8.0, 100 mM NaCl, 1 mM TCEP, 1 mM ATP, and 2 mM MgCl_2_. Samples were measured in triplicates using a BioRad CFX96Touch RT-PCR System.

### Fluorescence Anisotropy.

The binding affinity of the C-terminal 35 amino acids of Akt1 to Akt1^ΔC^ was determined by reverse titration of 200 to 250 μM Akt1^ΔC^ in a buffer containing 100 nM fluorescein-labeled peptide with the sequence SMEAVDSERRPHFPQFSYSASGTA (unphosphorylated). The obtained binding curves were fit with a one-site binding model to estimate the binding affinity. Fluorescence anisotropy was measured on a Perkin-Elmer LS50 fluorimeter with λ_*ex*_ = 500 nm and λ_*em*_ = 518 nm, at 20 °C in 20 mM Tris, pH 8.0, 100 mM NaCl, and 1 mM TCEP. Each concentration of Akt1^ΔC^ was measured 50 times with an integration time of 1 s and the mean plotted. The error bars represent the SD of the measurements. Three independent titrations were performed.

## Supplementary Material

Supplementary File

## Data Availability

Structure coordinates data have been deposited in the Protein Data Bank (7APJ).

## References

[1] B. D. Manning, A. Toker, AKT/PKB signaling: Navigating the network. Cell 169, 381–405 (2017). 28431241 10.1016/j.cell.2017.04.001 PMC5546324

[2] K. M. Siess, T. A. Leonard, Lipid-dependent Akt-ivity: Where, when, and how. Biochem. Soc. Trans. 47, 897–908 (2019). 31147387 10.1042/BST20190013 PMC6599160

[3] B. D. Manning, L. C. Cantley, AKT/PKB signaling: Navigating downstream. Cell 129, 1261–1274 (2007). 17604717 10.1016/j.cell.2007.06.009 PMC2756685

[4] D. A. Fruman., The PI3K pathway in human disease. Cell 170, 605–635 (2017). 28802037 10.1016/j.cell.2017.07.029 PMC5726441

[5] S. George. A family with severe insulin resistance and diabetes mellitus due to a missense mutation in AKT2. Science 304, 1325–1328 (2008). 10.1126/science.1096706 PMC2258004 15166380

[6] M. Frech., High affinity binding of inositol phosphates and phosphoinositides to the pleckstrin homology domain of RAC/protein kinase B and their influence on kinase activity. J. Biol. Chem. 272, 8474–8481 (1997). 9079675 10.1074/jbc.272.13.8474

[7] S. R. James., Specific binding of the Akt-1 protein kinase to phosphatidylinositol 3,4,5-trisphosphate without subsequent activation. Biochem. J. 315, 709–713 (1996). 8645147 10.1042/bj3150709 PMC1217264

[8] C. C. Thomas, M. Deak, D. R. Alessi, D. M. F. van Aalten, High-resolution structure of the pleckstrin homology domain of protein kinase b/akt bound to phosphatidylinositol (3,4,5)-trisphosphate. Curr. Biol. 12, 1256–1262 (2002). 12176338 10.1016/s0960-9822(02)00972-7

[9] N. Kannan, N. Haste, S. S. Taylor, A. F. Neuwald, The hallmark of AGC kinase functional divergence is its C-terminal tail, a cis-acting regulatory module. Proc. Natl. Acad. Sci. U.S.A. 104, 1272–1277 (2007). 17227859 10.1073/pnas.0610251104 PMC1783090

[10] W. J. Oh., mTORC2 can associate with ribosomes to promote cotranslational phosphorylation and stability of nascent Akt polypeptide. EMBO J. 29, 3939–3951 (2010). 21045808 10.1038/emboj.2010.271 PMC3020639

[11] V. Facchinetti., The mammalian target of rapamycin complex 2 controls folding and stability of Akt and protein kinase C. EMBO J. 27, 1932–1943 (2008). 18566586 10.1038/emboj.2008.120 PMC2486276

[12] D. R. Alessi., 3-Phosphoinositide-dependent protein kinase-1 (PDK1): Structural and functional homology with the Drosophila DSTPK61 kinase. Curr. Biol. 7, 776–789 (1997). 9368760 10.1016/s0960-9822(06)00336-8

[13] D. Stokoe., Dual role of phosphatidylinositol-3,4,5-trisphosphate in the activation of protein kinase B. Science 277, 567–570 (1997). 9228007 10.1126/science.277.5325.567

[14] D. D. Sarbassov, D. A. Guertin, S. M. Ali, D. M. Sabatini, Phosphorylation and regulation of Akt/PKB by the rictor-mTOR complex. Science 307, 1098–1101 (2005). 15718470 10.1126/science.1106148

[15] J. Yang., Molecular mechanism for the regulation of protein kinase B/Akt by hydrophobic motif phosphorylation. Mol. Cell 9, 1227–1240 (2002). 12086620 10.1016/s1097-2765(02)00550-6

[16] J. Yang., Crystal structure of an activated Akt/protein kinase B ternary complex with GSK3-peptide and AMP-PNP. Nat. Struct. Biol. 9, 940–944 (2002). 12434148 10.1038/nsb870

[17] T. O. Chan., Resistance of Akt kinases to dephosphorylation through ATP-dependent conformational plasticity. Proc. Natl. Acad. Sci. U.S.A. 108, E1120–E1127 (2011). 22031698 10.1073/pnas.1109879108 PMC3219155

[18] K. Lin., An ATP-site on-off switch that restricts phosphatase accessibility of Akt. Sci. Signal. 5, ra37 (2012). 22569334 10.1126/scisignal.2002618

[19] S. Lu., The mechanism of ATP-dependent allosteric protection of Akt kinase phosphorylation. Structure 23, 1725–1734 (2015). 26256536 10.1016/j.str.2015.06.027 PMC7734571

[20] I. Lučić., Conformational sampling of membranes by Akt controls its activation and inactivation. Proc. Natl. Acad. Sci. U.S.A. 115, E3940–E3949 (2018). 29632185 10.1073/pnas.1716109115 PMC5924885

[21] N. Chu., Akt kinase activation mechanisms revealed using protein semisynthesis. Cell 174, 897–907.e14 (2018). 30078705 10.1016/j.cell.2018.07.003 PMC6139374

[22] N. Balasuriya., Genetic code expansion and live cell imaging reveal that Thr308 phosphorylation is irreplaceable and sufficient for Akt1 activity. J Biol. Chem. 293, 10744–10756 (2018). 29773654 10.1074/jbc.RA118.002357 PMC6036199

[23] M. Ebner, I. Lučić, T. A. Leonard, I. Yudushkin, PI(3,4,5)P_3_ engagement restricts Akt activity to cellular membranes. Mol. Cell 65, 416–431.e6 (2017). 28157504 10.1016/j.molcel.2016.12.028

[24] V. Calleja, M. Laguerre, P. J. Parker, B. Larijani, Role of a novel PH-kinase domain interface in PKB/Akt regulation: Structural mechanism for allosteric inhibition. PLoS Biol. 7, e17 (2009). 19166270 10.1371/journal.pbio.1000017 PMC2628406

[25] V. Calleja, M. Laguerre, B. Larijani, 3-D structure and dynamics of protein kinase B-new mechanism for the allosteric regulation of an AGC kinase. J. Chem. Biol. 2, 11–25 (2009). 19568789 10.1007/s12154-009-0016-8 PMC2682354

[26] V. Calleja., Intramolecular and intermolecular interactions of protein kinase B define its activation in vivo. PLoS Biol. 5, e95 (2007). 17407381 10.1371/journal.pbio.0050095 PMC1845162

[27] W.-I. Wu., Crystal structure of human AKT1 with an allosteric inhibitor reveals a new mode of kinase inhibition. PLoS One 5, e12913 (2010). 20886116 10.1371/journal.pone.0012913 PMC2944833

[28] J.-M. Lapierre., Discovery of 3-(3-(4-(1-aminocyclobutyl)phenyl)-5-phenyl-3H-imidazo[4,5-b]pyridin-2-yl)pyridin-2-amine (ARQ 092): An orally bioavailable, selective, and potent allosteric AKT inhibitor. J. Med. Chem. 59, 6455–6469 (2016). 27305487 10.1021/acs.jmedchem.6b00619

[29] Weisner J., Preclinical efficacy of covalent-allosteric AKT inhibitor Borussertib in combination with Trametinib in KRAS-mutant pancreatic and colorectal cancer. Cancer Res. 79, 2367–2378 (2019). 30858154 10.1158/0008-5472.CAN-18-2861

[30] S.-L. Liu., Quantitative lipid imaging reveals a new signaling function of phosphatidylinositol-3,4-bisphophate: Isoform- and site-specific activation of Akt. Mol. Cell 71, 1092–1104.e5 (2018). 30174291 10.1016/j.molcel.2018.07.035 PMC6214670

[31] N. Jethwa., Endomembrane PtdIns(3,4,5)P3 activates the PI3K-Akt pathway. J. Cell Sci. 128, 3456–3465 (2015). 26240177 10.1242/jcs.172775

[32] N. Chu., The structural determinants of PH domain-mediated regulation of Akt revealed by segmental labeling. eLife 9, e59151. (2020). 32744507 10.7554/eLife.59151 PMC7438110

[33] S. A. Forbes., COSMIC: Exploring the world’s knowledge of somatic mutations in human cancer. Nucleic Acids Res. 43, D805–D811 (2015). 25355519 10.1093/nar/gku1075 PMC4383913

[34] M. J. Lindhurst., A mosaic activating mutation in AKT1 associated with the Proteus syndrome. N. Engl. J. Med. 365, 611–619 (2011). 21793738 10.1056/NEJMoa1104017 PMC3170413

[35] J. H. Lee., De novo somatic mutations in components of the PI3K-AKT3-mTOR pathway cause hemimegalencephaly. Nat. Genet. 44, 941–945 (2012). 22729223 10.1038/ng.2329 PMC4417942

[36] D. Alcantara., Mutations of AKT3 are associated with a wide spectrum of developmental disorders including extreme megalencephaly. Brain 140, 2610–2622 (2017). 28969385 10.1093/brain/awx203 PMC6080423

[37] J. D. Carpten., A transforming mutation in the pleckstrin homology domain of AKT1 in cancer. Nature 448, 439–444 (2007). 17611497 10.1038/nature05933

[38] K. E. Landgraf, C. Pilling, J. J. Falke, Molecular mechanism of an oncogenic mutation that alters membrane targeting: Glu17Lys modifies the PIP lipid specificity of the AKT1 PH domain. Biochemistry 47, 12260–12269 (2008). 18954143 10.1021/bi801683k PMC2919500

[39] C. Parikh., Disruption of PH-kinase domain interactions leads to oncogenic activation of AKT in human cancers. Proc. Natl. Acad. Sci. U.S.A. 109, 19368–19373 (2012). 23134728 10.1073/pnas.1204384109 PMC3511101

[40] T. Okuzumi., Inhibitor hijacking of Akt activation. Nat. Chem. Biol. 5, 484–493 (2009). 19465931 10.1038/nchembio.183 PMC2783590

[41] E. K. Han., Akt inhibitor A-443654 induces rapid Akt Ser-473 phosphorylation independent of mTORC1 inhibition. Oncogene 26, 5655–5661 (2007). 17334390 10.1038/sj.onc.1210343

[42] M. Ahmad, W. Gu, T. Geyer, V. Helms, Adhesive water networks facilitate binding of protein interfaces. Nat. Commun. 2, 1–7 (2011). 10.1038/ncomms1258 21448160

[43] A. C. Dar, T. E. Dever, F. Sicheri, Higher-order substrate recognition of eIF2alpha by the RNA-dependent protein kinase PKR. Cell 122, 887–900 (2005). 16179258 10.1016/j.cell.2005.06.044

[44] C. Kim, C. Y. Cheng, S. A. Saldanha, S. S. Taylor, PKA-I holoenzyme structure reveals a mechanism for cAMP-dependent activation. Cell 130, 1032–1043 (2007). 17889648 10.1016/j.cell.2007.07.018

[45] H. Song., Phosphoprotein-protein interactions revealed by the crystal structure of kinase-associated phosphatase in complex with phosphoCDK2. Mol. Cell 7, 615–626 (2001). 11463386 10.1016/s1097-2765(01)00208-8

[46] J. R. Haling., Structure of the BRAF-MEK complex reveals a kinase activity independent role for BRAF in MAPK signaling. Cancer Cell 26, 402–413 (2014). 25155755 10.1016/j.ccr.2014.07.007

[47] A. K. Patel, R. P. Yadav, V. Majava, I. Kursula, P. Kursula, Structure of the dimeric autoinhibited conformation of DAPK2, a pro-apoptotic protein kinase. J. Mol. Biol. 409, 369–383 (2011). 21497605 10.1016/j.jmb.2011.03.065

[48] X. Zhang., Kinetic mechanism of AKT/PKB enzyme family. J. Biol. Chem. 281, 13949–13956 (2006). 16540465 10.1074/jbc.M601384200

[49] D. Balzano., Alternative activation mechanisms of protein kinase B trigger distinct downstream signaling responses. J. Biol. Chem. 290, 24975–24985 (2015). 26286748 10.1074/jbc.M115.651570 PMC4599004

[50] N. A. Warfel, M. Niederst, A. C. Newton, Disruption of the interface between the pleckstrin homology (PH) and kinase domains of Akt protein is sufficient for hydrophobic motif site phosphorylation in the absence of mTORC2. J. Biol. Chem. 286, 39122–39129 (2011). 21908613 10.1074/jbc.M111.278747 PMC3234737

[51] S. Choi., Agonist-stimulated phosphatidylinositol-3,4,5-trisphosphate generation by scaffolded phosphoinositide kinases. Nat. Cell Biol. 18, 1324–1335 (2016). 27870828 10.1038/ncb3441 PMC5679705

[52] M. T. Kunkel, Q. Ni, R. Y. Tsien, J. Zhang, A. C. Newton, Spatio-temporal dynamics of protein kinase B/Akt signaling revealed by a genetically encoded fluorescent reporter. J. Biol. Chem. 280, 5581–5587 (2005). 15583002 10.1074/jbc.M411534200 PMC2913970

[53] B. Ananthanarayanan, M. Fosbrink, M. Rahdar, J. Zhang, Live-cell molecular analysis of Akt activation reveals roles for activation loop phosphorylation. J. Biol. Chem. 282, 36634–36641 (2007). 17928291 10.1074/jbc.M706227200

[54] K. Sasaki, M. Sato, Y. Umezawa, Fluorescent indicators for Akt/protein kinase B and dynamics of Akt activity visualized in living cells. J. Biol. Chem. 278, 30945–30951 (2003). 12773546 10.1074/jbc.M212167200

[55] T. A. Leonard, Reply to Agarwal: Activity against nuclear substrates is not necessarily mediated by nuclear Akt. Proc. Natl. Acad. Sci. U.S.A. 115, E6101–E6102 (2018). 29907610 10.1073/pnas.1808882115 PMC6142218

[56] X. Zhou., Location-specific inhibition of Akt reveals regulation of mTORC1 activity in the nucleus. Nat. Commun. 11, 1–14 (2020). 33257668 10.1038/s41467-020-19937-w PMC7705703

[57] E. Pardon., A general protocol for the generation of Nanobodies for structural biology. Nat. Protoc. 9, 674–693 (2014). 24577359 10.1038/nprot.2014.039 PMC4297639

[58] R. Karlsson, P. S. Katsamba, H. Nordin, E. Pol, D. G. Myszka, Analyzing a kinetic titration series using affinity biosensors. Anal. Biochem. 349, 136–147 (2006). 16337141 10.1016/j.ab.2005.09.034

[59] A. J. McCoy., *Phaser* crystallographic software. J. Appl. Cryst. 40, 658–674 (2007). 19461840 10.1107/S0021889807021206 PMC2483472

[60] P. Emsley, K. Cowtan, Coot: Model-building tools for molecular graphics. Acta Crystallogr. D Biol. Crystallogr. 60, 2126–2132 (2004). 15572765 10.1107/S0907444904019158

[61] P. V. Afonine., Towards automated crystallographic structure refinement with *phenix.refine*. Acta Crystallogr. D Biol. Crystallogr. 68, 352–367 (2012). 22505256 10.1107/S0907444912001308 PMC3322595

[62] M. V. Petoukhov, P. V. Konarev, A. G. Kikhney, D. I. Svergun, *ATSAS* 2.1 – Towards automated and web-supported small-angle scattering data analysis. J. Appl. Cryst. 40, s223–s228 (2007).

[63] H. C. Jubb., Arpeggio: A web server for calculating and visualising interatomic interactions in protein structures. J. Mol. Biol. 429, 365–371 (2017). 27964945 10.1016/j.jmb.2016.12.004 PMC5282402

