# Supplementary material for: Structure of autoinhibited Akt1 reveals mechanism of PIP3-mediated activation
Source: Proc Natl Acad Sci U S A. 2021 Aug 12;118(33):e2101496118. doi: 10.1073/pnas.2101496118 (PMC8379990; doi:10.1073/pnas.2101496118)
Supplement: Supplementary File [file pnas.2101496118.sapp.pdf]

PNAS
www.pnas.org

Supplementary Information for

Structure of autoinhibited Akt1 reveals mechanism of PIP_3-mediated activation.

Linda Truebestein[1,2], Harald Hornegger[1,2], Dorothea Anrather[3], Markus Hartl[3], Kaelin D. Fleming[4], Jordan T.B. Stariha[4], Els Pardon[5,6], Jan Steyaert[5,6], John E. Burke[4,7] and Thomas A. Leonard[1,2,8]

Correspondence: Thomas A. Leonard
Email: thomas.leonard@meduniwien.ac.at

This PDF file includes:

Supplementary text
Figures S1 to S6
Tables S1 to S2
Supplementary Materials and Methods
SI References

Supplementary Information Text

Supplementary Materials and Methods

Protein purification

Briefly, Sf9 cells were resuspended in lysis buffer (50 mM Tris, pH 8, 100 mM NaCl, 50 mM NaF, 1 mM TCEP (tris carboxyethylphosphine), 20mM imidazole, with protease inhibitor cocktail

containing 1 mM PMSF, 100 μM bestatin, 14 μM E-64, 10 μM pepstatin A, and 1 μM phosphoramidon, for 1 hr. The lysate was centrifuged at 39 000 × g for 40 min and the supernatant loaded onto a 5 mL HisTrap column (GE Healthcare) equilibrated in Ni_A buffer (50 mM Tris pH 8, 100 mM NaCl, 50 mM NaF, 1 mM TCEP, 20 mM imidazole). The column was washed with Ni_A buffer (50 mM Tris pH 8, 100 mM NaCl, 50 mM NaF, 1 mM TCEP, 20 mM imidazole) and protein eluted from the column with a 0-500 mM gradient of imidazole. Peak fractions were incubated with TEV protease (made in-house) overnight in order to remove the tag. TEV-cleaved protein was further purified by high-resolution anion exchange chromatography on a MonoQ (GE Healthcare) column equilibrated in Q_A buffer (50 mM Tris pH 8, 1 mM EDTA, 1 mM TCEP). Peak fractions were collected, concentrated, and purified by size exclusion chromatography on a Superdex 200 10/30 column (GE Healthcare) equilibrated in 20 mM Tris, pH 8.0, 100 mM NaCl, 1 mM TCEP. Di-phosphorylated $Akt1^{2P}$ was prepared according to the protocol of Chu et al. (1) with the following changes: Akt1 1-459-intein-CBD was co-expressed with PDK1 using a pFastBac Dual vector in baculovirus-infected Sf9 insect cells. Cells were lysed in 50 mM Tris, pH 7.4, 100 mM NaCl, 50 mM NaF, 1 mM sodium orthovanadate, 2 mM β-glycerophosphate, 1 mM EDTA, 1 mM TCEP and protease inhibitors (Sigma P8849). After centrifugation to remove insoluble material, the fusion protein was bound to chitin resin, washed in lysis buffer and cleaved with 300 mM MESNA (sodium mercaptoethylsulfonate) overnight. The cleaved Akt1, bearing a C-terminal thioester, was then purified by Q-anion exchange chromatography and reacted overnight with a peptide corresponding to the missing C-terminal 30 amino acids of Akt1 (CVDSERRPHFPQFSYSASGTA) phosphorylated on S473 (bold underline). The corresponding ligation product was subsequently incubated with purified GST-PDK1 in vitro in buffer supplemented with 1 mM ATP and 2 mM $MgCl_2$ for 3 h at 4°C. Finally, the phosphorylated ligation product was further purified by high-resolution MonoQ anion exchange chromatography and verified by intact mass spectrometry. Fractions containing $Akt1^{2P}$ phosphorylated on T308 and S473 were concentrated in 20 mM Tris, pH 7.4, 100 mM NaCl, 50 mM NaF and 1 mM TCEP for use in downstream experiments. Akt1 1-445-intein-CBD was co-expressed with PDK1 in Sf9 insect cells. Cells were lysed in 50 mM Tris, pH 8.0, 150 mM NaCl, 1 mM sodium orthovanadate, 10 mM β-glycerophosphate, 2 mM sodium pyrophosphate, 2 mM benzamidine, 2 mM EDTA, 1 mM TCEP (tris carboxyethylphosphine) and protease inhibitor cocktail containing 1 mM PMSF, 100 μM bestatin, 14 μM E-64, 10 μM pepstatin A, and 1 μM phosphoramidon. After centrifugation the supernatant was bound to cellulose in binding buffer (50 mM Tris, pH 8.0, 150 mM NaCl, 2 mM benzamidine, 2 mM EDTA, 1 mM TCEP, 0.25% CHAPS) for 1 hour at 4°C. The flow through of the cellulose purification step was collected and bound to chitin beads in binding buffer for 2 hours at 4°C. The washed chitin beads were cleaved over night at 4°C in binding buffer containing 150 mM MESNA (sodium mercaptoethylsulfonate). Cleaved Akt1, bearing a C-terminal thioester, was further purified by Q-anion exchange chromatography (Q_A buffer: 50 mM Tris pH 8.0, 2 mM EDTA, 1 mM TCEP, Q_B buffer: buffer Q_A + 1 M NaCl) and concentrated for use in downstream protein ligation or protein crystallization. $Akt1^{3P}$ phosphorylated on T308, T450 and S473 was prepared by culturing Sf9 cells co-expressing Akt1 S475A T479R and human PDK1 for 72 h prior to the addition of the Akt inhibitor A-443654 at 1 μM for 18 h and okadaic acid (100 nM) for 60 min prior to harvesting. $Akt1^{3P}$ was purified as described for $Akt1^{1P}$ with the following changes. The protein was purified using a StrepTrap column (GE Healthcare) in lysis buffer containing 50 mM Tris, pH 7.4, 100 mM NaCl, 50 mM NaF, 1 mM sodium orthovanadate, 10 mM β-glycerophosphate, 2 mM sodium pyrophosphate, 2 mM benzamidine, 1 mM EDTA, 1 mM TCEP and protease inhibitor cocktail containing 1 mM PMSF, 100 μM bestatin, 14 μM E-64, 10 μM pepstatin A, 1 μM phosphoramidon. The protein was washed with buffer $Strep_A$ (50 mM Tris, pH 7.4, 100 mM NaCl, 50 mM NaF, 1 mM EDTA, 1 mM TCEP) and eluted in buffer $Strep_B$ ($Strep_A$ with addition of 2.5 mM D-desthiobiotin). Peak fractions were pooled and subjected to overnight cleavage with TEV protease and further purification by high-resolution MonoQ anion exchange chromatography and SEC as described for Akt^{1P}. SEC was performed in 20 mM Tris, pH 8.0, 100 mM NaCl, 50 mM NaF, 1 mM TCEP and 1 mM EDTA. The phosphorylation state was verified by intact mass spectrometry and the phosphorylated residues identified by tandem mass spectrometry. Nanobody CA13841 (NB41) was expressed in E. coli WK6 cells from a pMESy4 plasmid in LB +

0.5% glucose and 2 mM $MgCl_2$. Nanobody expression was induced with 1 mM IPTG and the cultures grown overnight at 28 °C. Cells were lysed by osmotic shock at 4°C in 15 mL TS buffer (200mM Tris pH 8, 500 mM sucrose). After 1 h, 30 mL of TS/4 buffer (50 mM Tris pH 8, 125 mM sucrose) were added and stirred for 45 min. The cell lysate was centrifuged for 30 min with 7500 g at 4 °C. NB41 was purified via immobilized metal affinity chromatography followed by size exclusion chromatography (Superdex 200 10/300) in 20 mM Tris pH 7.5, 100 mM NaCl and 1mM TCEP.

Intact mass spectrometry

Intact protein samples were diluted in H_2O and up to 100 ng protein were loaded on an Aeris Widepore C4 column (3.6 µm particle size, dimensions 2.1 × 150 mm; Phenomenex) or an XBridge Protein BEH C4 column (2.5 µm particle size, dimensions 2.1 mm X 150 mm; Waters) using a Dionex Ultimate 3000 HPLC system (Thermo Fisher Scientific) with a working temperature of 50 °C, 0.1% formic acid (FA) as solvent A, 80% acetonitrile, 0.08% FA as solvent B. Proteins were separated on a 6 min gradient from 10 to 80% solvent B at a flow rate of 300 µL/min and analysed on a Synapt G2-Si coupled via a ZSpray ESI source (Waters). Data were recorded with MassLynx V 4.1 (Waters) and analyzed using the MaxEnt 1 process to reconstruct the uncharged average protein mass.

Tandem mass spectrometry

The reduced protein was denatured in 4 M urea, 50 mM ammonium bicarbonate (ABC) and alkylated with 20 mM iodoacetamide for 30 min at room temperature in the dark. The sample was diluted with 50 mM ABC down to 1 M urea and then digested overnight using mass-spec-grade trypsin (Promega) at 37 °C or sequencing-grade endoproteinase GluC (Roche) at 25 °C. For the pepsin (Promega) digest, the protein sample was buffered with 50 mM ABC and after alkylation the protein sample was acidified with 10mM HCl to a pH < 3, and digested with pepsin for 3 hours at 37 °C. Protease-protein ratio was 1:30 for all digests. The digestion was stopped by adding trifluoroacetic acid to a final concentration of 1% and the peptides were desalted using custom-made C18 stagetips (2). The peptides were separated on an Ultimate 3000 RSLC nano-flow chromatography system (Thermo Fisher Scientific), using a pre-column for sample loading (PepMapAcclaim C18, 2 cm × 0.1 mm, 5 µm) and a C18 analytical column (PepMapAcclaim C18, 50 cm × 0.75 mm, 2 µm, both Dionex-Thermo Fisher Scientific), applying a linear gradient from 2 to 35% solvent B (80% acetonitrile, 0.08% FA acid; solvent A: 0.1% FA) at a flow rate of 230 nl/min over 60 min. Eluting peptides were analysed on a Q Exactive HF-X Orbitrap mass spectrometer equipped with a Proxeon nanospray source (Thermo Fisher Scientific), operated in data-dependent mode. Survey scans were obtained in a scan range of 375–1500 m/z, at a resolution of 60000 at 200 m/z and an AGC target value of 3E6. The 8 most intense ions were selected with an isolation width of 1.6 Da, fragmented in the HCD cell at 28% collision energy and the spectra recorded at a target value of 1E5 and a resolution of 30000. Peptides with a charge of +1 were excluded from fragmentation, the peptide match and exclude isotope features were enabled and selected precursors were dynamically excluded from repeated sampling. Raw data were processed using the MaxQuant software package (version 1.6.0.16, http://www.maxquant.org/) (3) and searched against a custom database containing the sequences of the Akt1 constructs, as well as all Spodoptera spp. protein sequences available in Uniprot and the Bombyx mori reference proteome (www.uniprot.org) plus the sequences of common contaminants. The search was performed with full trypsin specificity and a maximum of two missed cleavages, C-terminal cleavage to glutamate and aspartate for the GluC digests or unspecific cleavage for pepsin digests. Carbamidomethylation of cysteine residues was set as fixed, oxidation of methionine, protein N-terminal acetylation and phosphorylation of serine, threonine and tyrosine as variable modifications—all other parameters were set to default.

Results were filtered at a false discovery rate of 10% at the peptide and protein level. Spectra of phosphorylated Akt1 peptides were validated manually. All proteomics data have been deposited in the PRIDE repository with the identifier PXD022044 (4).

HDX-MS

Lipid Vesicle Preparation

Lipid vesicles containing 20% cholesterol (Sigma: 47127-U), 30% egg-yolk phosphatidylcholine (PC) (Avanti: 840051C), 15% brain phosphatidylserine (PS) (Avanti: 840032C), 35% egg-yolk phosphatidylethanolamine (PE) (Sigma: P6386) and lipid vesicles containing 20% cholesterol, 25% PC, 15% PS, 35% PE, and 5% phosphatidylinositol 3,4,5-trisphosphate (PIP_3) (Echelon: P-3916) were prepared by combining lipid components dissolved in organic solvent, vigorously mixing, and evaporating the solvent under a stream of argon gas to produce an even lipid film layer. The lipid film was desiccated under a vacuum for 45 minutes and resuspended at 4 mM in lipid buffer (20 mM Hepes pH 7.5, 100 mM KCl) by vortexing. The resuspended lipid vesicle solutions were sonicated for 10 minutes, subjected to ten freeze-thaw cycles, and sonicated again for 5 minutes. The vesicles were then snap-frozen in liquid nitrogen and stored at -80°C.

HDX-MS sample preparation

For HDX reactions comparing Akt^{1P} and $Akt^{\Delta C}$, exchange was carried out in a 50 µl reaction containing 30 picomoles of protein, either Akt^{1P} or $Akt^{\Delta C}$. To initiate hydrogen-deuterium exchange, 5 µL of either protein was incubated with 45 µL of D_2O buffer solution (10 mM HEPES pH 7.5, 100 mM NaCl, 96.2% D_2O) for five time points (3s on ice, 3s, 30s, 300s, 3000s at room temperature) to give a final concentration of 86.5% D_2O. Exchange was terminated by the addition of ice-cold acidic quench buffer at a final concentration 0.6 M guanidine-HCl and 0.9% formic acid, and samples were immediately frozen in liquid nitrogen and stored at -80°C. HDX reactions comparing phosphorylated $Akt1^{3P}$ with or without PIP_3 and $Akt1^{3P}$ with or without ATPγS were conducted in 25 µl reaction volumes with a final $Akt1^{3P}$ amount of 17.4 pmol. Three conditions were tested: $Akt1^{3P}$ with 0% PIP_3 vesicles (20% cholesterol, 30% PC, 15% PS, 35% PE), $Akt1^{3P}$ with 5% PIP_3 vesicles (20% cholesterol, 25% PC, 15% PS, 35% PE, 5% PIP_3), and $Akt1^{3P}$ with 0% PIP_3 vesicles and 1 mM final ATPγS. The final lipid vesicle concentration for all conditions was 400 µM. Exchange was carried out for four time points (3s, 30s, 300s and 3000s at room temperature). Prior to the addition of D_2O, the protein was incubated with lipid vesicles for 30 minutes at room temperature. Hydrogen deuterium exchange was initiated by the addition of 20 µl of D_2O buffer solution without ATPγS (20 mM HEPES pH 7.5, 100 mM NaCl, 2.5 mM Tris pH 7.4, 81.5% D_2O) or 20 µl of D_2O buffer solution with ATPγS (20 mM HEPES pH 7.5, 100 mM NaCl, 1.25 mM ATPγS, 81.5% D_2O) to 5µl of the protein-lipid solution, to give a final concentration of 65.2% D_2O. Exchange was terminated by the addition of ice-cold quench buffer to give a final concentration 0.6 M guanidine-HCl and 0.9% formic acid and samples were frozen in liquid nitrogen and stored at -80°C. All experiments were carried out in independent triplicate. All HDX-MS data have been deposited in the PRIDE repository with identifier PXD022061 (4).

Protein digestion and MS/MS data collection

Protein samples were rapidly thawed and injected onto an integrated fluidics system containing a HDx-3 PAL liquid handling robot and climate-controlled chromatography system (LEAP Technologies), a Dionex Ultimate 3000 UHPLC system, as well as an Impact HD QTOF Mass

spectrometer (Bruker). The protein was run over either one (at 10°C) or two (at 10°C and 2°C) immobilized pepsin columns (Applied Biosystems; Poroszyme Immobilized Pepsin Cartridge, 2.1 mm x 30 mm; Thermo-Fisher 2-3131-00; Trajan; ProDx protease column, 2.1 mm x 30 mm PDX.PP01-F32) at 200 mL/min for 3 minutes. The resulting peptides were collected and desalted on a C18 trap column (Acquity UPLC BEH C18 1.7mm column (2.1 x 5 mm); Waters 186003975). The trap was subsequently eluted in line with a C18 reverse-phase separation column (Acquity 1.7 mm particle, 100 x 1 mm^2 C18 UPLC column, Waters 186002352), using a gradient of 5-36% B (Buffer A 0.1% formic acid; Buffer B 100% acetonitrile) over 16 minutes. Mass spectrometry experiments were acquired over a mass range from 150 to 2200 m/z using an electrospray ionization source operated at a temperature of 200C and a spray voltage of 4.5 kV.

Peptide identification

Peptides were identified from the non-deuterated samples of Akt^{1P} or $Akt1^{3P}$ using data-dependent acquisition following tandem MS/MS experiments (0.5 s precursor scan from 150-2000 m/z; twelve 0.25 s fragment scans from 150-2000 m/z). MS/MS datasets were analysed using PEAKS7 (PEAKS), and peptide identification was carried out by using a false discovery-based approach, with a threshold set to 1% using a database of purified proteins and known contaminants. The search parameters were set with a precursor tolerance of 20 ppm, fragment mass error 0.02 Da, charge states from 1-8, leading to a selection criterion of peptides that had a -10logP score of 21.7.

Mass Analysis of Peptide Centroids and Measurement of Deuterium Incorporation

HD-Examiner Software (Sierra Analytics) was used to automatically calculate the level of deuterium incorporation into each peptide. All peptides were manually inspected for correct charge state, correct retention time, appropriate selection of isotopic distribution, etc. Deuteration levels were calculated using the centroid of the experimental isotope clusters. Results are presented as relative levels of deuterium incorporation and the only control for back exchange was the level of deuterium present in the buffer (86.5% and 65.2%). Differences in exchange in a peptide were considered significant if they met all three of the following criteria: ≥6% change in exchange, ≥0.4 Da difference in exchange, and a p value <0.01 using a two tailed student t-test. The raw HDX data are shown in two different formats. The raw peptide deuterium incorporation graphs for a selection of peptides with significant differences are shown in Supplementary Figure 4, with the raw data for all analyzed peptides in the source data. To allow for visualization of differences across all peptides, we utilized number of deuteron difference (#D) plots. These plots show the total difference in deuterium incorporation over the entire H/D exchange time course, with each point indicating a single peptide. Samples were only compared within a single experiment and were never compared to experiments completed at a different time with a different final D_2O level. The data analysis statistics for all HDX-MS experiments are in Supplemental Table 2 according to the guidelines of Masson et al (5). Raw HDX-MS data was uploaded to the PRIDE repository with accession number PXD022061.

Nanobody generation against $Akt1^{1P}$

A llama was immunized six times with a total of 0.9 mg $Akt1^{1P}$ in the presence of adjuvant at weekly intervals. For the first 3 injections the immunogen was gently mixed with an equal volume of GERBU adjuvant which was injected subcutaneously. For the last 3 injections, $Akt1^{1P}$ was injected subcutaneously and separately an equal volume of GERBU was injected to locally boost

the immune system. Four days after the final boost, 100 ml of blood was collected in EDTA-coated blood tubes. Peripheral blood lymphocytes were isolated using Uni-SepMAXI+ tubes according to the manufacturer's instructions. Total RNA was prepared using the Qiagen RNeasy midi kit and cDNA was synthesized by the SuperScriptII reverse transcriptase using 50µg of total RNA. To amplify the variable domains of all immunoglobulin heavy chains (VHs and VHHs) from the cDNA two gene-specific primers (CALL001: GTCCTGGCTGCTCTTCTACAAGG; CALL002: GGTACGTGCTGTTGAACTGTTCC) were used. The ≈700-bp PCR fragment, representing the VHH fragments, was gel-purified by using the QIAquick gel extraction kit (QIAGEN) according to the manufacturer's instructions. The open reading frames of the nanobodies were amplified by PCR from this fragment (using primers EP229: CCTTGAGCTCTTCGGCACAGGTGC-AGCTGGTGGAGTCTGG and EP230: AGGACTGCTCTTCCACTGGAGACGG-TGACCTGGGT) and cloned as SapI digested fragments in a Golden Gate variant of pMESy4 (GenBank KF415192) to establish a pMESy4 library in TG1 cells of 5E8 independent nanobody clones bearing a C-terminal hexa-His tag and a CaptureSelect sequence tag (Glu-Pro-Glu-Ala).

Nanobody-displaying phage particles were rescued before each round of panning by adding helper phage; for this, six OD600 units of the (sub)library was inoculated into a baffled Erlenmeyer containing 60 ml of 2×TY medium supplemented with 100 µg/mL ampicillin and 2% (w/v) glucose. Cells are grown at 37 °C and 200 rpm until the cells reached logarithmic phase (corresponding to an OD600 of 0.5) after which 10 ml of the log-grown cells were superinfected with 10 times more VCSM13 helper phage. After an incubation of 30 min, the cells were recovered and transferred to a new Erlenmeyer flask containing 60 ml of 2×TY medium supplemented with 100 µg/mL ampicillin, 25 µg/mL kanamycin and 2% (w/v) glucose. The overnight culture was centrifuged and phage present in the supernatant were precipitated by adding 10 ml of a 20% (w/v) PEG6000/2.5 M NaCl solution and keeping the tube on ice for at least 30 min. The phage particles were pelleted by centrifugation for 10 min at 3,200g and 4 °C. The supernatant was discarded and the precipitated phage particles were resuspended in ice-cold PBS.

To identify $Akt1^{1P}$ specific nanobodies, $Akt1^{DB}$ (in 20 mM Tris pH7.4, 100 mM NaCl, 1 mM TCEP) was captured on NeutrAvidin-coated Maxisorp plates and used as a target in the bio-panning. The $Akt1^{DB}$ containing wells as well as some blank wells were blocked with 4% of milk for 2 h. Nanobody-displaying phage particles were either used alone or were mixed with a 40-fold excess of $Akt1^{DA}$ before adding them to the $Akt1^{DB}$ wells. After a 1 h incubation time, the wells were washed 15 times to eliminate the aspecific phage and $Akt1^{1P}$ specific nanobodies were recovered by incubating each well with 100 µl of 0.25 mg/mL trypsin solution for 30 min at RT on a vibrating platform. After transferring the trypsin solution to a tube containing 5 µl of a 4 mg/ mL AEBSF solution to inhibit protease activity, the recovered phage particles were used to infect freshly grown TG1 cells and cells were grown ON in 2xTY supplemented with ampicillin (100 µg/mL) and 2% glucose. After two rounds of selection, cells from both selection rounds were plated out. Single colonies were grown ON at 37°C in 2xTY supplemented with ampicillin (100 µg/mL) and 2% glucose in a 96 well master plate. To prepare *E. coli* periplasmic extracts containing nanobodies, 10 µl of an overnight-grown master plate was inoculated in a 96-deep well plate containing 1 ml of 2×TY plus 100 µg/mL ampicillin and 0.1% (w/v) glucose per well. Plates were covered with a sterile gas-permeable adhesive seal and incubated for 3 h at 37 °C and 200 rpm. Nanobody expression was induced by adding IPTG (1mM) followed by shaking for 4–6 h at 37 °C and 200 rpm. Thereafter the cells were pelleted at 3,200g for 10 min, the supernatant was discarded and cell pellets were stored at −20 °C. The deep-well plate containing cell pellets was thawed for 15 min at RT, 100 µl of PBS was added to each well and incubated for 30 min on a vibrating platform. The plate was centrifuged for 10 min at 3,200g and 4 °C after which 20 µl of the supernatant was used to screen for target specificity via ELISA. For screening, 100 µl of a 6 µg/mL NeutrAvidin solution in 100 mM $NaHCO_3$ pH 8.3 was used to coat individual wells of a 96-well Maxisorp plate overnight at 4 °C. The next day, the wells were washed five times with PBS and 200 µl of blocking solution (4% skimmed milk in 20 mM Tris pH 7.4, 100 mM NaCl) was added to all wells to block the plate for 2 h. The plate was washed 5 times with 20 mM Tris pH 7.4, 100 mM NaCl and $Akt1^{DB}$ (in 20 mM Tris pH 7.4, 100 mM NaCl, 1 mM TCEP) was captured

on the NeutrAvidin-coated Maxisorp plates in 30 min. In parallel, half of the wells were incubated only with buffer to serve as a negative control. The plate was washed as before. 20µl of periplasmic extract of each clone was diluted 5-fold in 0.4% skimmed milk, 20 mM Tris pH 7.4, 100 mM NaCl and was added to an $Akt1^{DB}$ and blank well and incubated for 1 h. To detect nanobody binding to each well, the plate was washed 5 times and incubated with a His-tag–specific mouse antibody followed by 5 washes and an incubation with an anti-mouse-alkaline-phosphatase conjugate according to the manufacturer's instructions. To develop the colorimetric reaction the plate was washed 5 times and 100 µl of freshly made PNPP reagent (2 mg ml−1 4-nitrophenyl phosphate disodium salt hexahydrate in 100 mM Tris pH 9.5, 5 mM $MgCl_2$ and 100 mM NaCl) was added to the plates. The absorption was measured over different times at 405nm wavelength. Clones were defined as positive when the ratio of the signal between the $Akt1^{DB}$ and blank well was more than 2. In total 230 single clones were screened in an ELISA and about 90 positive clones were identified. The positive clones were sequenced and 22 different families with correct ORFs were found. A family is defined as a group of nanobody amino acid sequences with high similarity in the CDR3 sequence. NB41 was discovered in the condition where an excess of $Akt1^{DA}$ was added to select for those binders that are more specific for the $Akt1^{1P}$ conformation.

Supplementary Figures

Figure S1. Akt1 protein constructs used in this study.

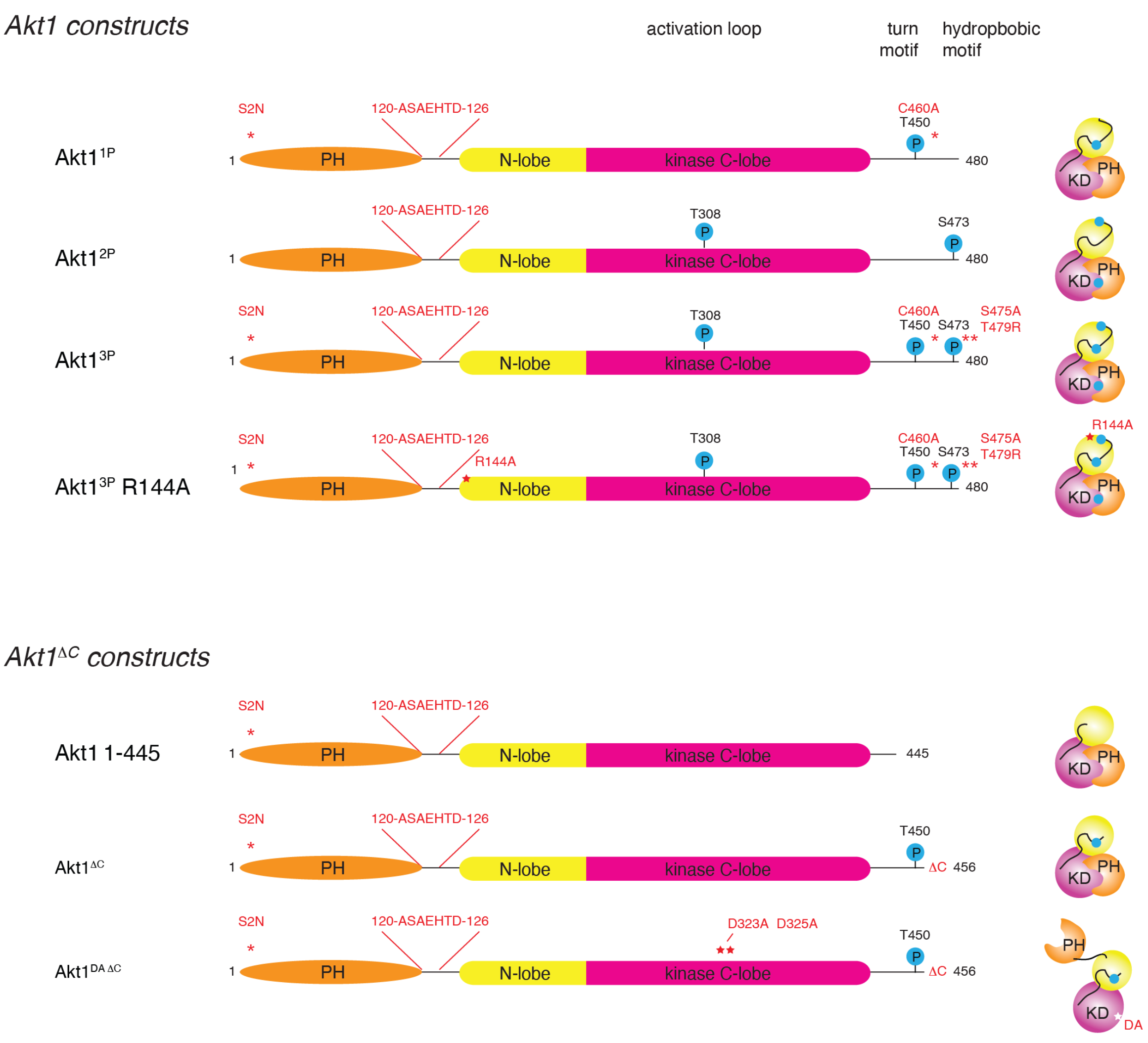

Fig. S1. Akt1 protein constructs used in this study.

Schematics of all Akt1 protein constructs used in this study, indicating domain boundaries, amino acid substitutions, protease cleavage sites, and phosphorylation sites. Numbering according to human Akt1. Blue spheres containing the letter P indicate the phosphorylation status of each site in each construct. Red stars indicate the position of the mutations introduced. The experimentally validated conformation of each species is indicated with a cartoon to the right of each construct.

Figure S2. The PIP_3 binding site is sequestered in autoinhibited Akt.

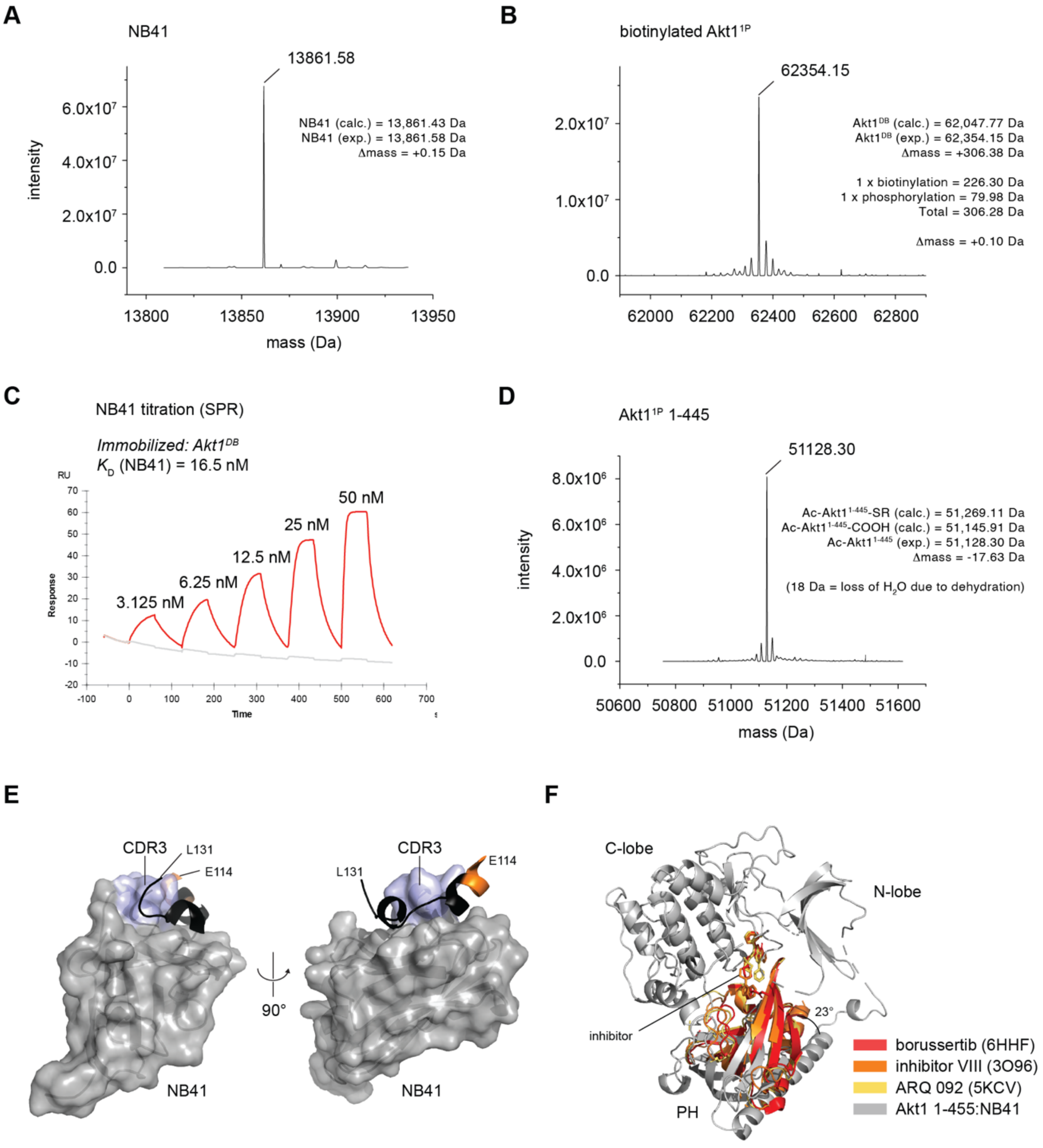

Fig. S2. The PIP_3 binding site is sequestered in autoinhibited Akt.

A. Intact mass spectrum of NB41.

B. Intact mass spectrum of biotinylated Akt1^{DB} used for immobilization in SPR experiments.

C. Binding affinity of NB41 for biotinylated Akt1^{DB} determined by single-cycle surface plasmon resonance (SPR).

D. Intact mass spectrum of Akt1 1-445 derived from MESNA-mediated intein cleavage. Close-up of the binding interface between NB41 and Akt1. The CDR3 antigen recognition loop is indicated in purple. Black, PH-kinase linker; orange, PH domain.

E. Superposition of Akt1 structures in complex with the allosteric inhibitors ARQ 092, borussertib and inhibitor VIII on the structure Akt1 1-445:NB41. Color of PH domain indicates allosteric inhibitor, kinase domain of autoinhibited Akt1 (only) shown for reference.

Figure S3. Akt1 prepared by protein semi-synthesis lacks a phosphorylated turn motif.

A

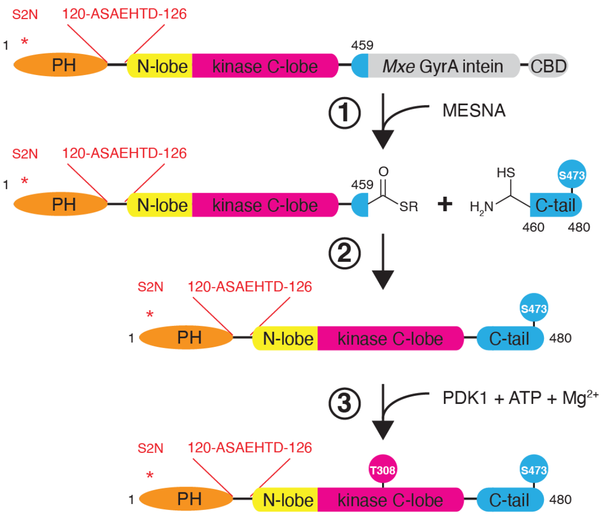

B

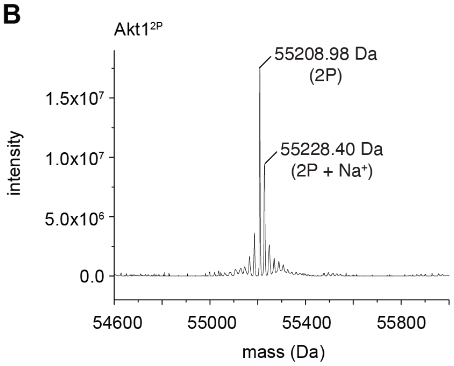

Mass (Da) (expected)	Mass (Da) (observed)	Δmass (Da)	Matching modifications
55003.1	55209.0	+205.9	+2P (T308, S473) +acetyl (M1)
	55228.4	+19.4	$+Na^+$

C

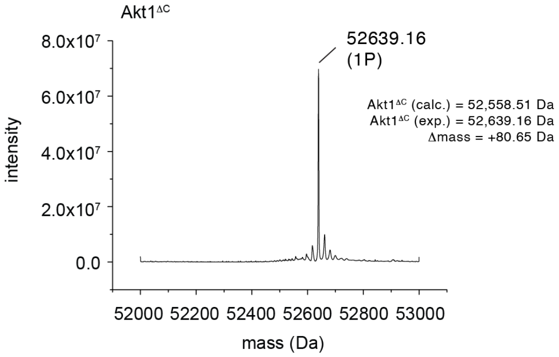

D

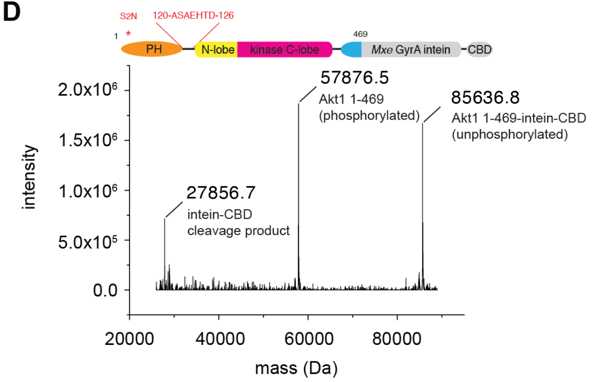

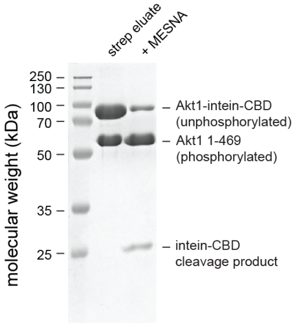

Mass (Da) (observed)	Mass (Da) (expected)	Matching species	Matching modifications	Δmass (Da)
27856.7	27859.2	intein-CBD cleavage product	none	+1.1
57876.5	57885.2	Akt1 1-469 (phosphorylated)	- N-terminal methionine + N-terminal acetyl + phosphate	+0.5
85636.8	85725.3	Akt1-intein-CBD (unphosphorylated)	- N-terminal methionine + N-terminal acetyl	+0.8

Fig. S3. Akt1 prepared by protein semi-synthesis lacks a phosphorylated turn motif.

A. Strategy for preparation of site-specifically phosphorylated Akt1 according to Chu et al (21).

B. Intact mass spectrometry of Akt1 after expressed protein ligation and *in vitro* phosphorylation with PDK1.

C. Intact mass spectrum of Akt1$^{\Delta C}$ (residues 1-456).

D. Intact mass spectrum of Akt1 1-469-intein-CBD fusion protein after elution from the Strep-Tactin affinity column. Table indicates observed and expected masses of the three major species together with the matching modifications that correspond to the observed masses. The final column indicates the mass difference between the expected and observed masses. Right: SDS-PAGE gel of the Strep-Tactin column eluate and its subsequent treatment with MESNA. Mass spectrometry was performed on the strep eluate (lane 2).

Figure S4. Phosphorylation does not override requirement for PIP_3.

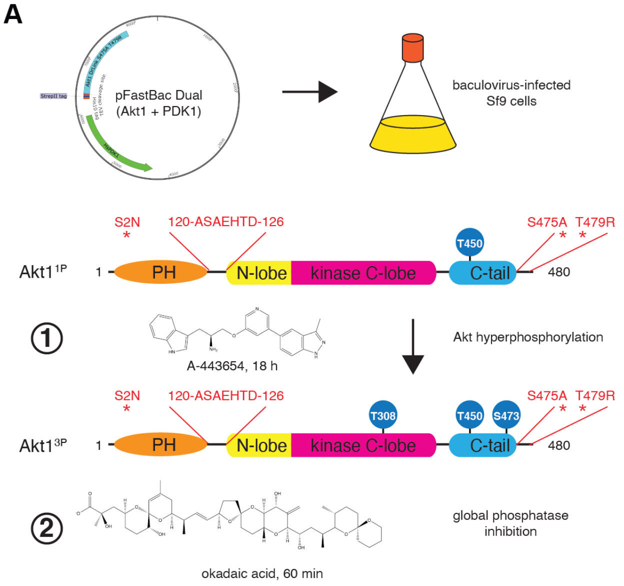

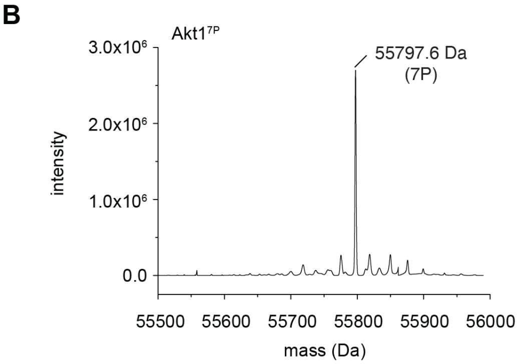

Mass (Da) (expected)	Mass (Da) (observed)	Δmass (Da)	PTMs	mod/base (% phos.) (phospho-sites)	
55237.4	55797.6	+560.2	+7P	S123	6.24 (86.2)
				T127	6.51 (86.7)
				S132	5.34 (84.2)
				T308	16.87 94.4)
				T450	12.68 (92.7)
				S473	3.26 (76.5)
				S477	3.55 (78.0)

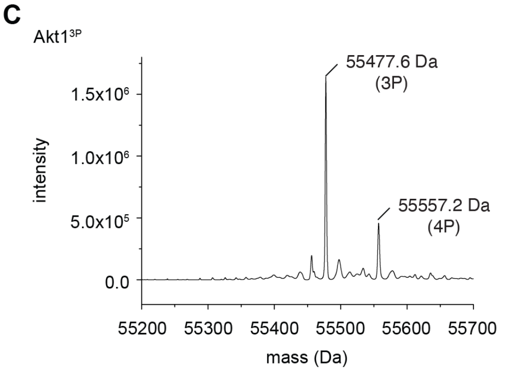

Mass (Da) (expected)	Mass (Da) (observed)	Δmass (Da)	Matching modifications
55237.4	55477.6	+240.2	+3P
	55557.2	+319.8	+4P

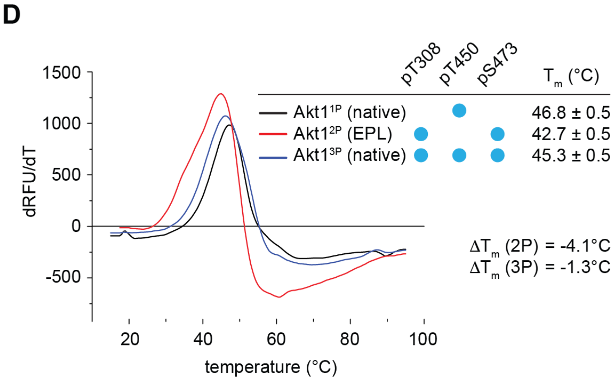

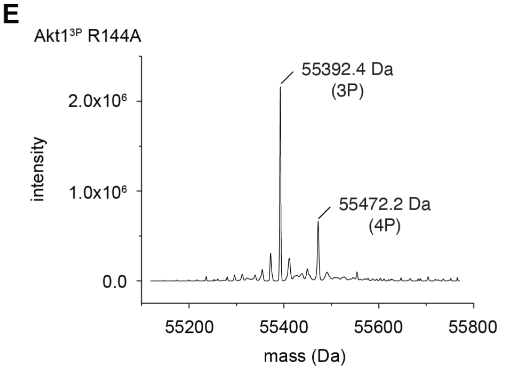

Mass (Da) (expected)	Mass (Da) (observed)	Δmass (Da)	Matching modifications
55152.3	55392.4	+240.1	+3P
	55472.2	+319.9	+4P

Fig. S4. Phosphorylation does not override requirement for PIP_3.

A. Strategy for the preparation of Akt1 phosphorylated on T308, T450 and S473.

B. Intact mass spectrum for Akt1^{7P} (seven phosphorylations) and phosphorylation profile of Akt1^{7P} determined by tandem mass spectrometry of a GluC digest.

C. Intact mass spectrometry of tris-phosphorylated Akt1^{3P} isolated by high-resolution anion-exchange chromatography.

D. Thermal stability analysis of Akt1^{1P}, Akt1^{2P}, and Akt1^{3P} by differential scanning fluorimetry. Black curve, mono-phosphorylated Akt1^{1P}; red curve, di-phosphorylated Akt1$^{2P;}$ blue curve, tris-phosphorylated Akt1^{3P}. (EPL = expressed protein ligation).

E. Intact mass spectrometry of tris-phosphorylated Akt^{3P} R144A isolated by high-resolution anion-exchange chromatography.

Figure S5. Phosphorylation alone does not drive Akt into an active conformation.

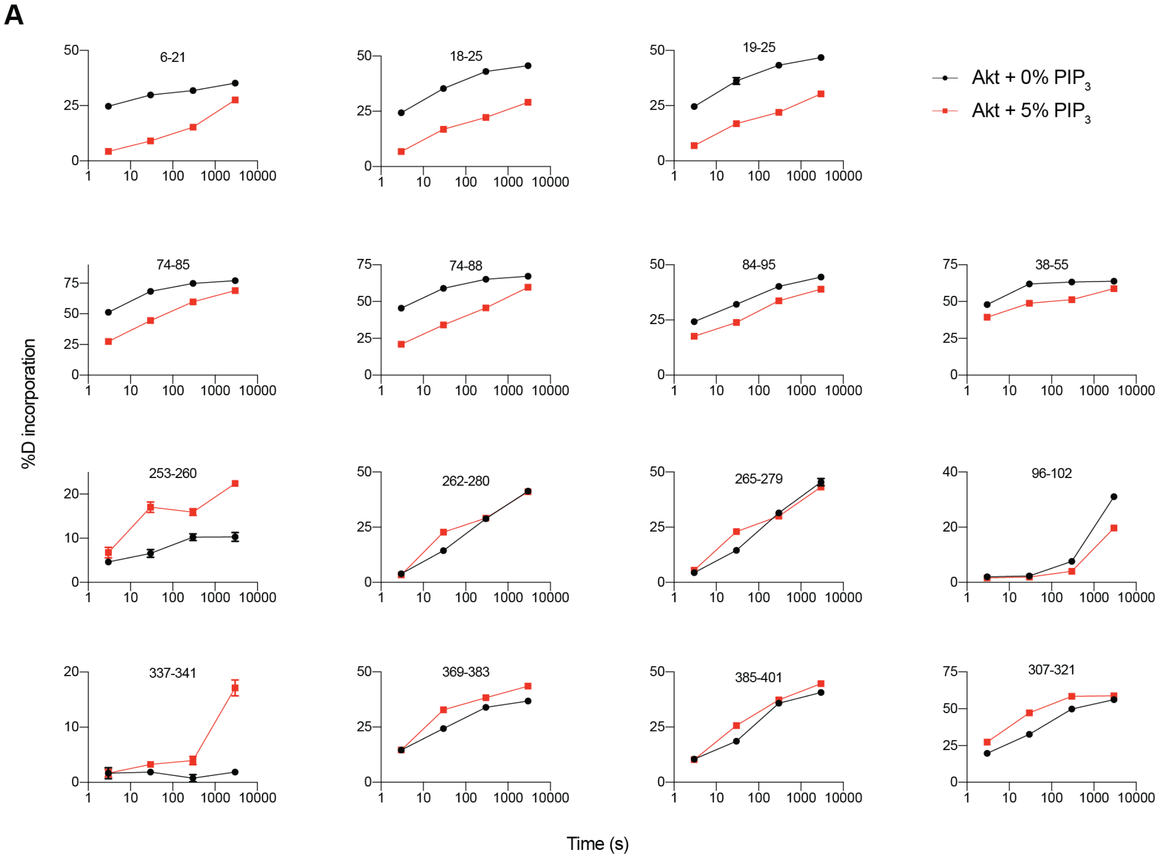

Fig. S5. Phosphorylation alone does not drive Akt into an active conformation.

A. HDX-MS plots for individual peptides in Akt1^{3P}. Black lines indicate deuterium incorporation in the presence of liposomes containing 0 mol % PI(3,4,5)P_3; red lines indicate the incorporation of deuterium in the presence of liposomes containing 5 mol % PI(3,4,5)P_3.

Figure S6. PIP$_3$-binding promotes Akt hydrophobic motif exposure.

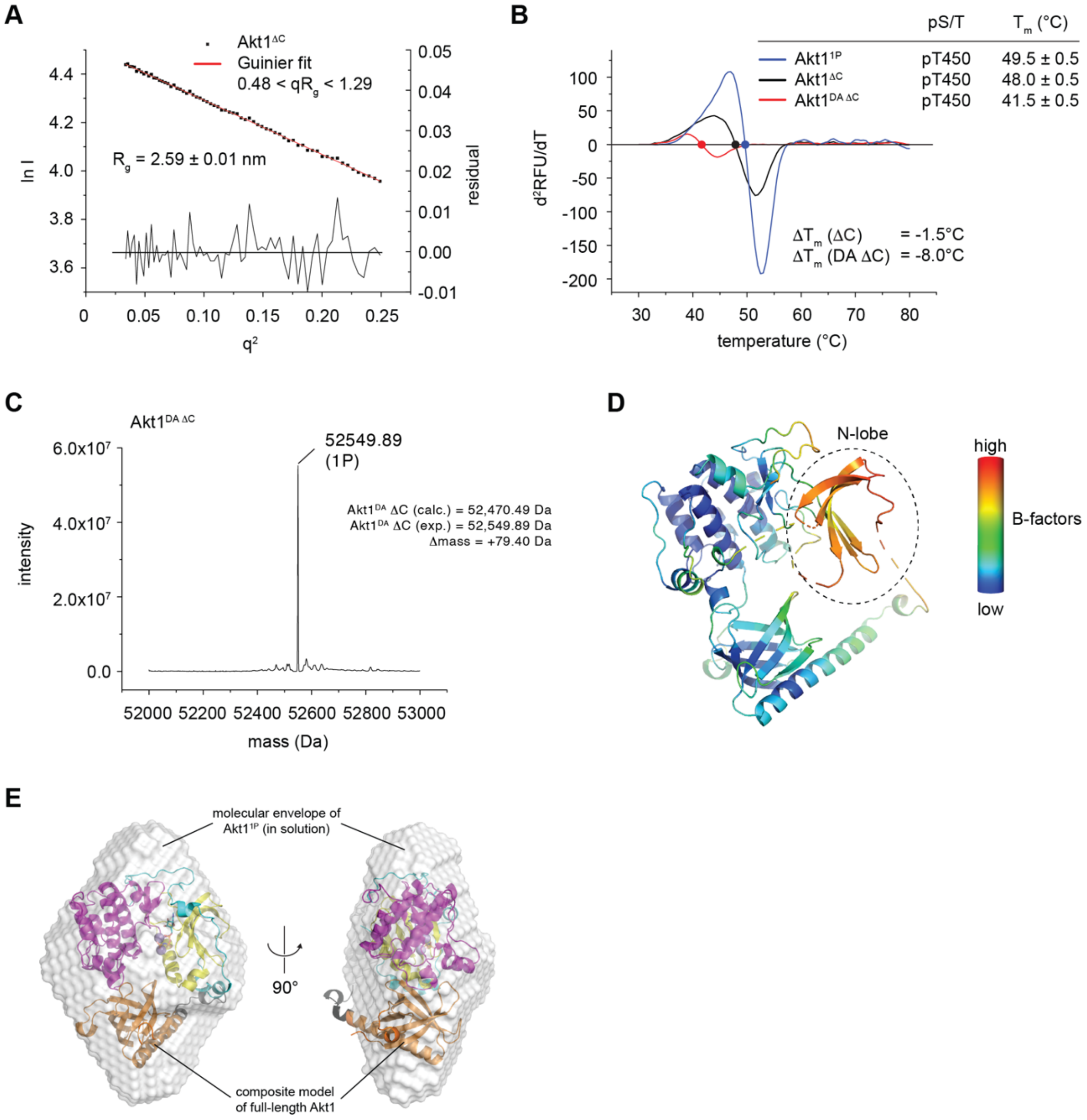

Fig. S6. PIP$_3$ binding promotes Akt hydrophobic motif exposure.

A. Guinier analysis of the small-angle X-ray scattering of Akt1$^{\Delta C}$.

B. Thermal stability analysis of Akt1^{1P} (blue), Akt1$^{\Delta C}$ (black), and Akt1$^{DA\ \Delta C}$ (red) by differential scanning fluorimetry.

C. Intact mass spectrum of Akt1$^{DA\ \Delta C}$.

D. Temperature factors (B-factors) mapped onto the structure of autoinhibited Akt1. Fit of composite model of full-length Akt1 into the *ab initio* molecular envelope of Akt1^{1P} derived from SAXS.

Table S1. Data Collection and Refinement Statistics

Data collection	***Native***
space group	$P2_12_12_1$
unit cell (*a, b, c* in Å)	70.00, 72.20, 120.17
wavelength (Å)	0.873
resolution range (Å)	45.60 – 2.05
observations	316,111
unique reflections	38,883
completeness (%)[a]	99.8 (99.1)
multiplicity	8.1 (7.9)
I/σ_I	9.7 (1.2)
R_{pim} (%)	4.9 (66.8)
$CC_{1/2}$	0.998 (0.658)
Structure refinement	
resolution range	45.60 – 2.05
reflections used	38,883
R-factor, R_{free}[b] (%)	21.10 (25.19)
modelled residues	
chain A (Akt1)	3-131, 140-153, 158-179, 197-288, 307-434
chain B (NB41)	3-119
waters	163
rms deviations	
bond lengths (Å)	0.002
bond angles (deg)	0.507
Ramachandran	
Residues in favored region	96.73 %
Residues in outlier region	0.2 %

[a] Values in parentheses refer to the highest resolution shell.

[b] R_{free} = free R-factor based on random 5 % of all data.

Table S2. HDX data collection and processing.

Data set	Akt1^{DrLink}	Akt1^{DrLink} ΔC	Akt1^{3P} + 0% PIP_3 vesicles	Akt1^{3P} + 5% PIP_3 vesicles	Akt1^{3P} + ATPγS
HDX reaction details	%D_2O=86.5%<br>pH $_{(read)}$=7.5<br>Temp=18˚C	%D_2O=86.5%<br>pH $_{(read)}$=7.5<br>Temp=18˚C	%D_2O=65.2%<br>pH $_{(read)}$=7.5<br>Temp=18˚C	%D_2O=65.2%<br>pH $_{(read)}$=7.5<br>Temp=18˚C	%D_2O=65.2%<br>pH $_{(read)}$=7.5<br>Temp=18˚C
HDX time course (seconds)	0.3, 30, 300, 3000	0.3, 30, 300, 3000	3, 30, 300, 3000	3, 30, 300, 3000	3, 30, 300, 3000
HDX controls	N/A	N/A	N/A	N/A	N/A
Back-exchange	Corrected based on %D_2O	Corrected based on %D_2O	Corrected based on %D_2O	Corrected based on %D_2O	Corrected based on %D_2O
Number of peptides	85	85	160	160	160
Sequence coverage	94.0%	94.0%	97.1%	97.1%	97.1%
Average peptide length/redundancy	Length=12.5<br>Redundancy= 2.2	Length=12.5<br>Redundancy= 2.2	Length=15.2<br>Redundancy= 5.1	Length=15.2<br>Redundancy= 5.1	Length=15.2<br>Redundancy= 5.1
Replicates	3 (0.3s, 3s, 30s), 2 (300s, 3000s)	3 (0.3s, 3s) , 2 (30s, 300s, 3000s)	3	3	3
Repeatability	Average StDev=0.3%	Average StDev=0.3%	Average StDev=0.6%	Average StDev=0.6%	Average StDev=0.6%
Significant differences in HDX	≥6% and ≥0.4 Da and unpaired t-test ≤0.01	≥6% and ≥0.4 Da and unpaired t-test ≤0.01	≥6% and ≥0.4 Da and unpaired t-test ≤0.01	≥6% and ≥0.4 Da and unpaired t-test ≤0.01	≥6% and ≥0.4 Da and unpaired t-test ≤0.01

Supplementary References

1. Chu N, Salguero AL, Liu AZ, Chen Z, Dempsey DR, Ficarro SB, Alexander WM, Marto JA, Li Y, Amzel LM, Gabelli SB, Cole PA (2018) Akt Kinase Activation Mechanisms Revealed Using Protein Semisynthesis. *Cell* 174(4):897-907.e14.

2. Rappsilber J, Mann M, Ishihama Y (2007) Protocol for micro-purification, enrichment, pre-fractionation and storage of peptides for proteomics using StageTips. *Nat Protoc* 2(8):1896–906.

3. Cox J, Mann M (2008) MaxQuant enables high peptide identification rates, individualized p.p.b.-range mass accuracies and proteome-wide protein quantification. *Nat Biotechnol* 26(12):1367–72.

4. Perez-Riverol Y, et al. (2019) The PRIDE database and related tools and resources in 2019: improving support for quantification data. *Nucleic Acids Res* 47(D1):D442–D450.

5. Masson GR, et al. (2019) Recommendations for performing, interpreting and reporting hydrogen deuterium exchange mass spectrometry (HDX-MS) experiments. *Nat Methods* 16(7):595–602.
